# Supplementary figures and images for: Quality of clinical assessment and management of sick children by Health Extension Workers in four regions of Ethiopia: A cross-sectional survey
Source: PLoS One. 2020 Sep 25;15(9):e0239361. doi: 10.1371/journal.pone.0239361 (PMC7518593; doi:10.1371/journal.pone.0239361)

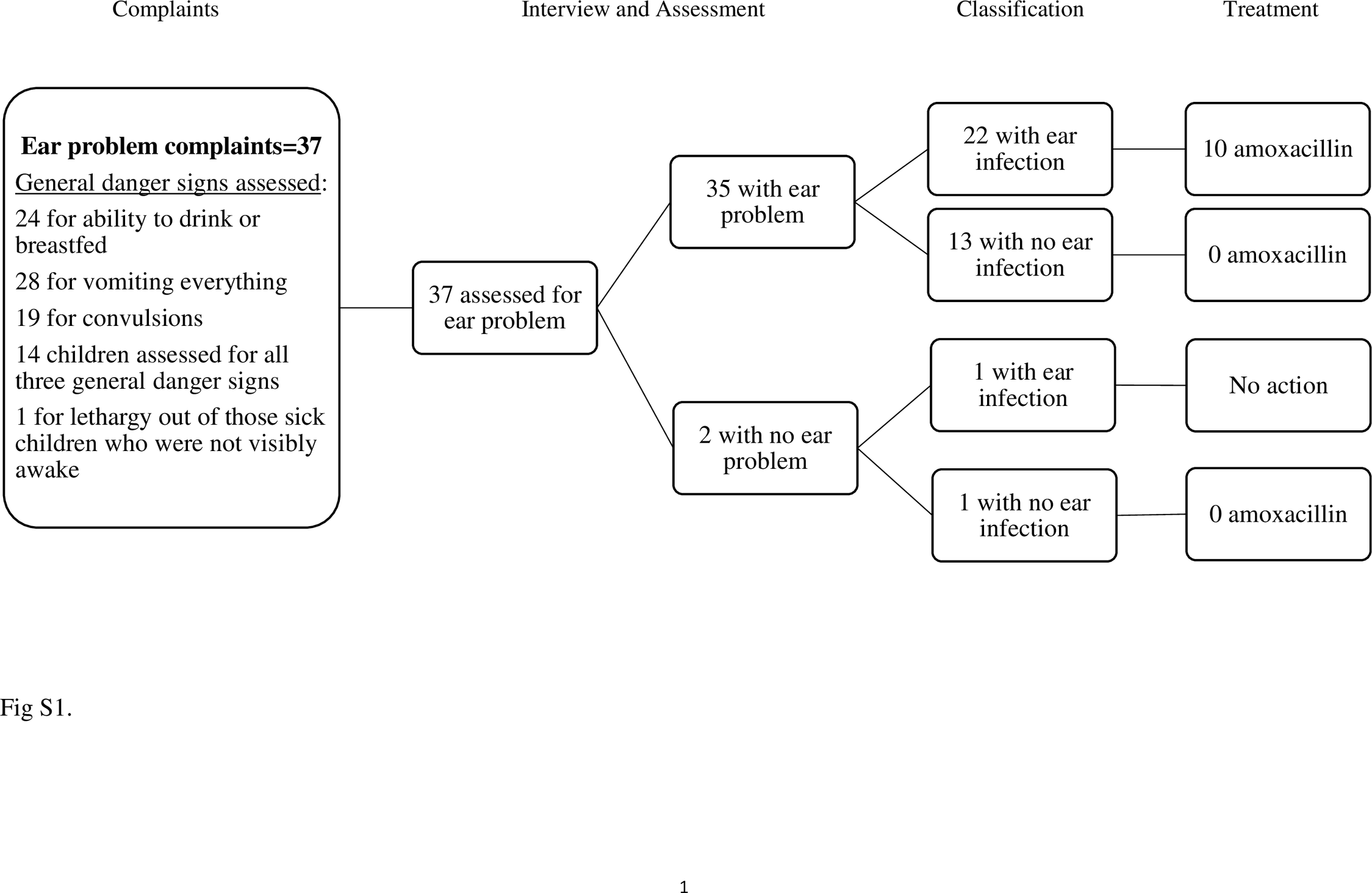

Supplement: S1 Fig — (TIF) [file pone.0239361.s001.tif]
