# Supplementary material for: Quality of clinical assessment and management of sick children by Health Extension Workers in four regions of Ethiopia: A cross-sectional survey
Source: PLoS One. 2020 Sep 25;15(9):e0239361. doi: 10.1371/journal.pone.0239361 (PMC7518593; doi:10.1371/journal.pone.0239361)
Supplement: S1 Table — (DOCX) [file pone.0239361.s002.docx]

S1 Table.

| Indicator | Sick children with one or more complaints  N=620  n(%)[95% CI] | Complaints of cough or difficulty of breathing  N=359  n(%)[95% CI] | Complaints of diarrhea  N=224  n(%)[95% CI] | Complaints of fever  N=163  n(%)[95% CI] | Complaints of ear problem  N=37  n(%)[95% CI] |
| --- | --- | --- | --- | --- | --- |
| Proportion of sick children with complaints of assessed for: |  |  |  |  |  |
| Bipedal edema | 228(37)  [33.0-40.6%] | 137(38)  [33.2-43.3%] | 88(39)  [33.1-45.8%] | 64(39)  [32.0-47.0%] | 7(19)  [8.70-33.9%] |
| Visible severe wasting (in children <6 months) | 35(47)^1^  [35.6-57.9%] | 26(55)^2^  [41.0-69.0%] | 15(48)^3^  [31.3-65.7%] | 77(45)^4^  [24.6-66.7%] | One sick child out of two |
| Palmar pallor | 314(51 )  [46.7-54.6%] | 193(54) [48.6-58.9%] | 113(50)  [43.9-57.0%] | 83(51)  [43.3-58.6%] | 12(32)  [18.9-48.6%] |
| Vaccination status | 455(73)  [69.8-76.8%] | 271(76) [70.8-79.7%] | 168(75)  [69.0-80.0%] | 119(73)  [65.8-79.4%] | 24(65)  [48.6-74.2%] |
| Vitamin A status | 366(59)  [55.1-62.9%] | 221(62) [56.4-66.5%] | 132(59)  [52.0-65.0%] | 110(67)  [60.0-74.3%] | 22(59)  [43.2-78.9%] |

**^*^** Physical danger signs included visible severe wasting, bipedal edema and palmar pallor (anemia)

^1^N=75, ^2^N=47, ^3^N=31, ^4^N=20
