# Supplementary material for: Quality of clinical assessment and management of sick children by Health Extension Workers in four regions of Ethiopia: A cross-sectional survey
Source: PLoS One. 2020 Sep 25;15(9):e0239361. doi: 10.1371/journal.pone.0239361 (PMC7518593; doi:10.1371/journal.pone.0239361)
Supplement: S1 Box — (DOCX) [file pone.0239361.s003.docx]

S1 Box.

1. Hygiene and environmental sanitation
   1. Excreta disposal
   2. Solid and liquid waste disposal
   3. Water supply and safety measures
   4. Food hygiene and safety measures
   5. Healthy home environment
   6. Control of insects and rodents
   7. Personal hygiene
2. Family health service
   1. Maternal and child health
   2. Family planning
   3. Immunization
   4. Adolescent reproductive health
   5. Nutrition
3. Disease prevention and control
   1. TB, HIV/AIDS and other STI prevention and control
   2. Malaria prevention and control
   3. First aid and emergency measures
4. Health education and communication (this is part of all of the packages)
