# Supplementary material for: Quality of clinical assessment and management of sick children by Health Extension Workers in four regions of Ethiopia: A cross-sectional survey
Source: PLoS One. 2020 Sep 25;15(9):e0239361. doi: 10.1371/journal.pone.0239361 (PMC7518593; doi:10.1371/journal.pone.0239361)
Supplement: S1 File — (ZIP) [file pone.0239361.s004.zip › S File/ObservationQuest_Afan Oromo Version.pdf]

Sakata'iinsa Hojjettoota Fayyaa

**100**	**Eenyummeessaa addaa tajaajila kennaa/HEF (ganda/dhaabbata fayyaa/koodii tajaajila kennaa)**	|__|__| / |__|__| / |__|__|
**101**	**Enyummeessaa addaa daa'imaa (ganda/dhaabbata fayyaa/koodii daa'imaa)**	|__|__| / |__|__| / |__|__| / |__|__|
**102**	**Guyyaa**	|__||__|/|__||__|/|__||__| gg / jj / bb
**103**	**Naannoo**	|________________________________|
**104**	**Godina**	|________________________________|
**105**	**Aanaa**	|________________________________|
**106**	**Ganda**	|________________________________|
**107**	**Koodii Kilaasterii**	|__|__|
**108**	**Laatiitiwudii GPS**	|__|__| |__|__| |__|__| DIG DAQ SEK
**109**	**Loongiitiwudii GPS**	|__|__| |__|__| |__|__| DIG DAQ SEK
	**Elevation**	|__|__|__|__|
**110**	**Maqaa dhaabbata fayyaa**	|_______________________________|
**111**	**Koodii dhaabbata fayyaa**	|__|__|
**112**	**Koodii HEF**	/__/__/ Lakka addaa
**113**	**Gosa tajaajila kennaa**	1=Hojjetaa fayyaa kutaa <5 gadi keessa hojjetu<br>2=Hojjettuu Ekisteenshinii Fayyaa
**114**	**Saala hojjetaa fayyaa**	1=Dhiira; 2=Dhalaa
116_01	**Guyyaa dhaloota HEF**	GG |___|__| JJ|___|__| BBBB |___|__|___|__|
116-02	**Akka HEF, sadarkaa oggumma keeti meeqa?**	1 = Sadarkaa 1ffa<br>2 = Sadarkaa 2ffa<br>3 = Sadarkaa 3ffa<br>4 = Sadarkaa 4ffa
116_03	**Oggumma HEFtin, hangam tajajiltee? (Tajajilaa gandaa bira keenite dabaltee waggaa meeqa tajajiltee?). Waggaa 1 gaddi yoo ta'e, ji'a tajajiltee barressii**	|___||___| Waggaa<br>|___||___| Ji'a
116_04	**Oggumma HEFtin, k/fayyaa kana kessatti hangam tajajiltee? Waggaa 1 gaddi yoo ta'e, ji'a tajajiltee barressii**	|___||___| Waggaa<br>|___||___| Ji'a
116_05	**Iddo jireenyaa keetti gandaa kana keessatti?**	1 = Eeyyee<br>2 =Lakki
116_06	**Wajjiraa gandaa mana jireenyaa siif keennera?**	1 = Eeyyee<br>2 =Lakki

**115**	**Hojjetaan/ttuun Ekisteenshinii fayyaa leenjii ICCM fudhateeraa/ teettii?**	1=Eeyyee; 2=Lakki-Gara 121 Darbi
**116**	**Bara leenjii ICCM fudhate/tte (bara barreessi, BBBB)**	|__|__|__|__|
**117**	**Ji'otaa 6'n darbaan kessatti hojjetun extension fayyaa marii hordoffii raawwiifi gorsa yaalaa (MHRGY) irratti hirmatetti?**	1=Eeyyee; 2=Lakki
**118**	**Ji'otaa 6'n darban kessatti hojjetun extension fayyaa degarsaa fi hordofii argatetti？**	1=Eeyyee; 2=Lakki
**119**	**Yoo lakk. 120 eyye ta'e： enyuutu yeroo dhiyoo kessaati degarsaa fi hordofii kenne?**	1=Wajjirra Egumsaa fayyaa Aanaa irraa<br>2=Buufataa fayyaa irraa<br>3=Dhabbataa mit-motummaa irra<br>4= Wajjirra Egumsaa fayyaa Aanaa fi Buufataa fayyaa irraa<br>5= Wajjirra Egumsaa fayyaa Aanaa fi Dhabbataa mit-motummaa irra<br>6= Wajjirra Egumsaa fayyaa Aanaa, Buufataa fayyaa fi Dhabbataa mit-motummaa<br>88=Kan biraa ibsi
**120**	**Yoo lakk. 120 eyyee ta'e; qabxillen arman gadii ilalamee jira. Isaa arman gadii dubbissi. hundaafuu 1=Eyyee; 2=Lakki**	|__|
122A	**Dhimma sakkata'uu ykn yallu dhukkubaa haqee (quufa) ta'uu danda'aa irratti walin mari'anne**	|__|
122B	**Dhimma sakkata'uu ykn yallu dhukubaa garaa kasaa irratti mari'anne**	|__|
122C	**Dhimma sakkata'uu ykn yallu dhukubaa busaa irratti mari'anne**	|__|
122D	**Dhimma sakkata'uu ykn yallu hiri'nna nyattaa irratti mari'anne**	|__|
122E	**Haala galmee illalu**	|__|
122F	**Haala itti fayaadamaa galmee：qixaafi guutu ta'uu isaa ilaalu/mirkanessu**	|__|
122G	**Dhiyyessii ilalchisee qajeelfamaa leenjii, Degaraa hojii fakkin, unkaa hojii addaa addaa jirachu isaa mirkanessu**	|__|
122H	**Dhiyyessii qajeeffamaa leenjii, Degaraa hojii fi unkaa hojii addaa addaa gochu**	|__|
122I	**<u>Halaa mamiilaa hojjettu extension fayyaa</u>**	|__|

	**gafatuu/tajaajiltu ilaalu)**	
122J	**Duubi deebbii barrefammaa hojii keetiifi kennu**	|__|
122K	**yoo sii kennameraa ta'ee duubi deebbii kee isaa dhummaa jira, ilaaluun danda'a**	|__|
**121**	**Maqaa daa'imaa**	|________________________________|
**122**	**Koodii daa'imaa**	|__|__|
**123**	**Saala daa'imaa**<br>**1=Dhiira; 2=Dhalaa**	|__|
**124**	**Umuriin daa'imaa meeqa?**<br>**Yoo waggaa 1 gadi ta'e, 00 jechuun lakkoofs ji'aa barreessi**	|__|__|waggaa<br>Yoo<1 : | __|__|ji'a
**125**	**Guyyaa dhaloota daa'imaa?**<br>**(gg/jj/bbbb)**	|__|__| / |__|__| / |__|__|__|__|
127_01	**Saala kunuunsaan daa'imaa**<br>**1=Dhiira; 2=Dhalaa**	|__|
127_02	**Harriroo kunuunsaan daa'imaa; daa'imaa wajjin qabu maal?**	1=Hadha daa'imaa<br>2=Abba daa'imma<br>3=Kan bira, addessi____
**126**	***Af-gaafataa*: HEF eeyyama kenneeraa/tti?**	1=Eeyyee; 2=Lakki. Yoo lakki ta'e ni dhuma
**127**	**Af-gaafataa: Warri/kunuunsaan daa'imaa mariin akka daawwatamu, gaaffii fi deebiin dhuma irratti akka ta'uu fi daa'imni irra deebi'ee akka qoratamuuf eeyyameeraa?**	1=Eeyyee; 2= Lakki. YOO LAKKI TA'E – DHUMA
**128**	**Qubeelee jalqabaa maqaa af-gaafataa**	|__|__|
**129**	**Guyyaa (gg/jj/bbbb)**	|__|__| / |__|__| / |__|__|__|__|

Moojula A: Cheekliistii Daawwannaa: Daa'ima (ji'a 2 – waggaa shanii)

Marii gidduu odoo hin seenin callisii daawwadhu. Waan argituu fi dhageessu galmeessi.

**130**	**Sa'a daawwiin itti eegale:**	|__|__|:|__|__|

Sakattii

**SABABA**		
	**Warri/kunuunsaan, daa'ima gara buufata fayyaa/keellaa fayyaa fiduuf sababa maalii dhihheesse/ssite? (Eeyyee kan ta'an hundaaf 1 barreessi)**	
**131**	**Hargansuu daddaffii/harganuu dadhabuu**	|__|
**132**	**Qufaa**	|__|
**133**	**Michii sombaa ykn Pnimoniyaa**	|__|
**134**	**Garaa kaasaa**	|__|
**135**	**Hooqqisiisuu/balaqqamsiisuu**	|__|
**136**	**Qaama gubaa**	|__|
**137**	**Busaa**	|__|
**138**	**Sochiin qaamaa tasaa fi to'annaan ala ta'e**	|__|
**139**	**Harma hodhuu ykn dhangala'aa fudhachuu dadhabuu**	|__|
**140**	**Rakkoo gurraa**	|__|
**141**	**Kan biroo (addeessi)**	|__|
**ULFAATINA**		
**142**	**Har'a: Hojjetaan/ttun Ekistenshinii fayyaa kun ykn kan biroon guyyaa har'aa ulfaatina daa'imaa madaaluun galmeessaniiruu?**	**1= Eeyyee; 2=Lakki-Gara 146 Darbi**
**143**	**Ulfaatinni daa'imaa hangami?** (Kiloograamaan)	|__|__| kg
**MALLATTOOLEE HAMOO**		
**144**	**HEF daa'imni harma hodhuu/dhangala'aa fudhachaa jiraachuu isaa gaafateeraa/ttii?**	1= Eeyyee; 2=Lakki-Gara 147 Darbi
146A	**Daaimma sun dhangala'aa fudhachuuf harma hodhuu dhadhabee jira?**	**1. Eyyee 2. Lakki**
**145**	**HEF daa'imni waan hunda akka hooqqisiisu/balaqqamu gaafateeraa/ttii?**	1=Eeyyee; 2= Lakki-Gara 148 Darbi
147A	**Daaimma sun waan hunduma balaqqsisaa jira?**	**1.Eyyee 2. Lakki**
**146**	**HEF daa'imni sochii qaamaa tasaa fi to'annaan alaa akka qabu gaafateeraa/ttii?**	1= Eeyyee; 2= Lakki-Gara 149 Darbi
148A	**Daa'imni sun sochii qaamaa tasaa fi to'annaan alaa ta'e qaba?**	**1.Eyyee 2. Lakki**
**147**	**Daa'imni haala mul'achuu danda'uun dammaqaa dhaa (FKN: taphachuu, kolfuu, humnaan boo'uu)?**	1= Eeyyee-Gara 151 Darbi<br>2= Lakki
**148**	**Yoo daa'imni haala mul'achuu danda'uun dammaqaa miti ta'e, HEF humna dhabuu ykn ofwallaaluu isaa mirkaneesseeraa/tti (daa'ima**	1= Eeyyee; 2= Lakki

	**dammaqsuu yaleeraa)?**	
150A	**Daa'imni sun ofwallaaleera?**	**1.Eyyee 2.Lakki**
**QUFAA/HARGANSUU**		
**149**	**HEF qufaan jiraachuu ykn harganuu dadhabuu daa'imaa gaafateeraa/ttii?**	1= Eeyyee; 2= Lakki -Gara 153 Darbi
**150**	**Daa'imni qufaa qabaa ykn harganuuu ni dadhabaa?**<br>1= Eeyyee; 2= Lakki-Gara 154 Darbi	|__|
**Yoo daa'imni rakkoo argaansuu ammo qabatee**<br>1= Eeyyee; 2= Lakki		
**151**	**HEF daa'ima irra qufaan ykn hargansu dadhabuu hangam irra akka ture gaafateeraa/ttii?**	|__|
**152**	**HEF hargansuu daa'imaa daqiiqaa 1 keessatti lakkaa'eera/ lakkoofteettii?-Yoo lakki ta'e Gara 152 Darbi**	|__|
**153**	**Yoo Eeyyee ta'e, daqiiqaa 1 keessatti meeqa lakkaa'e/lakkoofte?**	|__|__| bpm
**154**	**HEF qomni daa'imaa gadi dhooquu isaa ilaaleeraa/ilaalteettii?**	|__|
**155**	**HEF daa'imaa yeroo hargansuu sagalee jirachuu isaa mirkanesseraa?**	|__|
**GARAA KAASAA**		
**156**	**HEF daa'imni garaa kaasaa qabaachuu fi dhiisuu gaafateeraa/ ttii?**<br>1= Eeyyee; 2= Lakki-Gara 161 Darbi	|__|
**157**	**Daa'imni garaa kaasaaa qabaa?**<br>1= Eeyyee; 2= Lakki-Gara 161 Darbi	|__|
	**Yoo daa'imni GARAA KAASAA qaba ta'e;(1=Eyyee; 2=Lakki)**	
**158**	**HEF daa'mni yeroo meeqaaf garaa kaasaan akka irra ture gaafateeraa/ttii?**	|__|
**159**	**HEF booliiin daa'imaa dhiigaan kan wal make ta'uu gaafateeraa/ttii?**	|__|
**160**	**HEF daa'imni akka boqonnaa dhabuu fi bubbutamu qorateeraa?**	|__|
**161**	**HEF daa'imaaf dhangala'aa kenneeraa?**	|__|
**162**	**HEF gogaa garaa daa'imaa qimmiideeraa?**	|__|
**QAAMA GUBAA**		
**163**	HEF qaama gubaan akka jiru gaafateeraa/ilaaleeraa? (yoo ho'inni qaamaa safarameera ta'e galmee ilaaleeraa)?<br>1=Eeeyyee; 2=Lakki-Gara 176 Darbi	|__|
**164**	HEF ho'inni qaama daa'immaa safartetti? 1=Eeeyyee; 2=Lakki-Gara 164 Darbi	
**165**	Ho'inni qaama daa'imaa hangami? (digriidhaan)	|__|__|.|__|dig
**166**	Daa'imni qaama gubaa qabaa/qaba turee (sa'a 48 darbe keessatti)?<br>1=Eeeyyee; 2=Lakki-Gara 176 Darbi	|__|
	Yoo daa'imni* QAAMA GUBAA *qabaate, …<br>***(1=Eeyyee; 2= Lakki,)***	

**167**	HEF qaama gubaan daa'ima irra yeroo hangamiif akka ture gaafateeraa?	|__|
**168**	HEF qaamni gubaa kun yeroo hundaa akka jiru gaafateeraa?	|__|
**169**	HEF daa'imni goga mormaa (socho'uu hin danddeenye) qabaachuu isaa ilaaleeraa?	|__|
**170**	HEF gubbeen daa'imaa gadi dhooqaa ykn ol dhiita'aa ta'uu fi dhiisu isaa mirkaneesseeraa?	|__|
**171**	HEF daa'imni ji'a sadan darban keessatti gifiraan qabamu fi dhiisuu gaafateeraa? Ykn mallattolee gifiraa daa'ima irratti qorateeraa?[1]	|__|
**172**	HEF shifiin qaama daa'ima irra jiiraachuu fi dhiiisuu ilaaleeraa?	|__|
**173**	Ji'oota sadan darban keessatti daa'imni gifiraan qabamee tuee ykn mallattoleen gifiraa iratti mul'ataniru?	|__|
**174**	Hojjetaan fayyaa daa'imni madaa afaan keessaa akka qabu ilaaleeraa?	|__|
**175**	Hojjetaan fayyaa iji daa'imaa malaa qabaachuu ykn koorniyaan ijaa dimimmisaa'uu fi dhiisuu ilaaleeraa?	|__|
**176**	Hojjetaan fayyaa daa'imaaf RDT hojjeteeraa? Yoo lakki ta'e GARA 176 Darbi	|__|
**177**	Yoo RDTn qoratameera ta'e, firiin isaa maal ture?<br>1= Posatiiiva; 2= Negatiiva; 3= Kan hin hoojjennne/beekamne; 9= Hin beeku	|__|
**RAKKOO GURRAA**		
**178**	**HEF daa'imni rakkoo gurraa akka qabu gaafateeraa?**<br>1=Eeyyee; 2=Lakki-Gara 181 Darbi	|__|
**179**	**Daa'imni rakkoo guraa qabaa?**<br>1=Eeyyee; 2=Lakki-Gara 181 Darbi	|__|
	Yoo daa'imni rakkoo gurraa qabaate …(1=Eeyyee; 2=Lakki)	
**180**	**HEF daa'imni yeroo hangamiif rakkoo gurraa akka qabaate gaafateeraa?**	|__|
**181**	**HEF dhukkubbiin gurraa akka jiruu fi dhiisu gaafateeraa?**	|__|
**182**	**HEF dhangala'aa/malaan gurra keessaa ba'u jiraachu fi dhiisuu ilaaleeraa?**	|__|
**HANQINA NYAATAA**		
**183**	**HEF miilla lamaan gadi qabuun dhiitoon jiraachu fi dhiisuu ilaaleeraa?**<br>1=Eeyyee; 2=Lakki	|__|
**184**	**HEF hir'ina Qaamaa hamaae ta'e adda baaseeraa?**<br>1=Eeyyee; 2=Lakki	|__|
**185**	**HEF safara harka daa'imaa (MUAC) safareeraa/teettii?**<br>1=Eeyyee;<br>2=Lakki-Gara 185 Darbi<br>3=Hin ilalatuu(NA)	|__|

**186**	**Yoo eeyyee ta'e, safari harka daa'imaa (MUAC) meeqa (Santimetraan)?**<br>1=(<11); 2=(11.0-11.9); 3=(12.0 fi isaa ol)	|__|
**HIIR'INA DHIIGAA**		
**187**	**HEF Qamaa jijiramaa (palmar pallor) jirachuun isa ilaleraa?**<br>1=Eeyyee; 2=Lakki	|__|
**TALAALLII**		
**188**	**HEF kaardii talaallii daa'imaa ilaaluuf gaafateeraa?**<br>1=Eeyyee;<br>2=Lakki Gara 188 Darbi	|__|
**189**	**Kaardiin talaallii daa'imaa jiraa/argameeraa?**<br>1=Eeyyee; 2=Lakki	|__|
**190**	**HEF seenaa talaallii daa'imaa gaafateeraa/teettii?**<br>1=Eeyyee; 2=Lakki	|__|
**VITAMIN A**		
**191**	**HEF daa'imni coba vitamin A argatee akka beeku gaafateeraa/teettii?**<br>1=Eeyyee; 2=Lakki	|__|

Gosa Dhukkubaa

Gosa dhukkuba daa'imaa baruuf, galmee ICCM ilaali. Yoo gosi dhukkubaa galmee irratti hin galmoofne ta'e, gosooti dhukkuba daa'imaa maalfaa akka turan hojjetaa fayyaa gaafadhu.Hanga hojjetaan fayyaa gosoota dhukkubaa hunda tarrreessutti, "Gosa dhukkubaa kan biroon hoo?" jechuun gaafadhu.Waa'ee gosoota dhukkubaa tokko tokkoon hin gaafatin.

**192**	**HEF dhukkuba daa'imaaf gartuu tokko ykn isaa ol kenneeraa/teettii?**<br>1=Eeyyee; 2=Lakki	|__|
	Gosoota dhukkubaa armaan gaditti tarreeffamanii jiran hunda galmeessi	
**193**	Mallattoo hamaa tokkoo fi isaa ol (harma hodhuuu/dhangala'aa fudhachuu dadhabuu, waan hunda ballaqamuu, rom'uu qaamaa to'annaan ala ta'e, yoo qimmidame illee suuta socho'uu ykn tasumaa socho'uu dadhabuu	|__|
**194**	Michii sombaa hamaa ta'e/dhukkuba baay'ee cimaa ta'e	|__|
**195**	Michii sombaa	|__|
**196**	Michii sombaa hin qabu	|__|
**197**	Dhangala'a qaamaa (goga qaamaa) dhabuu cimaa ta'e	|__|
**198**	Goga qaamaa giddu-galeessa (dhangala'aa qaamaa dhabuu)	|__|
**199**	Goga qaamaa hin qabu	|__|
**200**	Garaa kaasaa cimaa fi turban lammaa ol ta'ee	|__|
**201**	Garaa kaasaa turban lammaa ol ta'ee	|__|
**202**	Garaa kaasaa dhiigaafi furrii walmakaa qabuu	|__|
**203**	Dhukkuba baa'yee cimaa fi ho'aa ta'e	|__|
**204**	Busaa	|__|
**205**	Gubaa qaamaa, busaa ta'uus dhiisuus ni danda'a	|__|
**206**	Gubaa qaamaa, busaa hin qabu	|__|
**207**	Gifira baay'ee hamaa fi wal-xaxaa ta'e	|__|
**208**	Gifiraa (Afan/Ija) wal-xaxaa ta'e	
**209**	Gifira	|__|
**210**	Dhibee gurraa hamaa fi ariifachiisaa ta'e	|__|
**211**	Dhibee gurraa yeroo dheeraa ture	|__|
**212**	Hanqina nyaataa cimaa	|__|
**213**	Hanqina nyaataa giddu-galeessa	|__|
**214**	Hir'ina dhiigaa hamaa	|__|
**215**	Hir'ina dhiigaa	|__|
**216**	Sadarkaan talaallii yeroo iaa kan eeggate miti (kan haara'e miti)	|__|
**217**	Talaallii barbaachisu (addeessi)	|__|
**218**	Sadarkaan/seenaan vitamin A kan haara'e miti	|__|
**219**	Kan biroo, addeessi ………………………	|__|
**220**	Kan biroo, addeessi………………………	|__|
**221**	Kan biroo, addeessi………………………	|__|

Yaalii

Hubachiisa: Af-gaafataan yeroo yaaaliin geggeefamutti, HEF tajaajilaa jiru waa'ee qorannoo godhee fi yaalii kenne gaafachuu ni danda'a; garuu kun kan ta'u yoo lameen yeroo mariitti hin ibsamne ta'e dha.

222	**HEF yaalii/talaallii ajajeeraa/kenneeraa?**<br>1=Eeyyee; 2=Lakki-Gara 276 Darbi	|__|
**ORS**		
223	**HEF ORS kenneeraa/kenniteettii?**<br>1=Eeyyee; 2=Lakki-Gara 228 Darbi ; 3=Ajajuu qofa	|__|
224	**ORS paakeetii meeqtu kenname? (lakkoofsaan)**	|__|
225	**HEF daa'imni keellaa fayyaa turee ORS akka fudhatu gorseeraa? (1= Eeyyee; 2= Lakki)**	|__|
226	**HEF ORSn akkamitti akka kennamu agarsiiseeraa? (1=Eeyyee; 2= Lakki)**	|__|
227	**HEF akkaataa ORSn itti kennamu waari/kunuunsaan daa'imaa deebisee akka itti argisiisu gaafateeraa? (1=Eeyyee; 2= Lakki)**	|__|
228	**HEF warri/kunuunsaan daa'imaa odoo keellaa fayyaatii hin ba'in dura doozii jalqabaa ORS daa'imaaf akka kennu gaafateeraa/ofii isaatii kenneeraafii? (1=Eeyyee; 2= Lakki)**	|__|
229	**HEF sadarkaa manaatti ORS ajajeeraa? (1=Eeyyee; 2= Lakki)**	|__|
**COARTEM (Qoricha busaa)**		
230	**HEF 'Koarteem' (Qoricha busaa) kenneeraa/kenniteettii?**	1=Eeyyee;<br>2=Lakki-Gara 235 Darbi<br>3= Ajajuu qofa
231	**Doozii tokkoo tokkoof 'Koarteem' ija meeqa?**	Kinini hanga guyyaati silaa|__|
232	**Guyyaatti si'a meeqaaf 'Koarteem' kennama?**	Bayina konini al tonnotti fudhatu|__|
233	**Guyyaa meeqaaf 'Koartem'n ajajame?**	Guyyaa |__|fi
234	**HEF akkaataa itti 'Koarteem' kennan agarsiiseeraa? (1= Eeyyee; 2= Lakki)**	|__|
235	**HEF warri/kunuunsaan daa'imaa akkaataa 'Koatem'n itti kennamu deebisee akka itti agarsiisu gaafateeraa? (1= Eeyyee; 2= Lakki)**	|__|
236	**HEF warri/kunuunsaan daa'imaa odoo keellaa fayyaatii hin ba'in dura doozii jalqabaa 'Koarteem' daa'imaaf akka kennu itti gaafateeraa/ofii isaatii kenneeraafii? (1=Eeyyee; 2= Lakki)**	|__|
**COTRIMOXAZOLE**		
237	**Hojjetaan fayyaa 'cotrimoxazole'kenneeraa?**<br>1=Eeyyee;<br>2=Lakki; Gara 244 Darbi<br>3= Ajajuu qofa	|__|
238	**Cotrimoxazole'n haala kamiin fudhatamuuf qophaa'e?**	|__|

	1=LIqimsaa ijaa daa'imaaf; 2=liqimsaa ijaa nama guddaaf; 3=shirooppii; 8=kan biroo	
239	**Doozii tokkoon tokkoof 'cotrimoxazole' ija meeqa? (lakkoofsaan)**	|__|.|__| tablets per dose
240	**Doozii tokkoon tokkoof 'cotrimoxazole' shirooppii ml hangami?**	|__| ml per dose
241	**Guyyaatti 'cotrimoxazole' si'a meeqaaf kennamuu qaba?**	|__| times per day
242	**Cotrimoxazole'n yeroo hangamiif/guyyaa meeqaaf ajajama?**	For |__| days
243	**Hojjetaan fayyaa akkaataa itti 'Cotrimoxazole'n kennan agarsiiseeraa? (1= Eeyyee; 2= Lakki)**	|__|
244	**Hojjetaan fayyaa warri/kunuunsaan daa'imaa akkaataa 'coatem'n itti kennamu deebisee akka itti agarsiisu gaafateeraa? (1= Eeyyee; 2= Lakki)**	|__|
245	**Hojjetaan fayyaa warri/kunuunsaan daa'imaa odoo keellaa fayyaatii hin ba'in dura doozii jalqabaa 'cotrimoxazole' daa'imaaf akka kennu gaafateeraa/ofii isaatii kenneeraafii? (1= Eeyyee; 2= Lakki)**	|__|
**ZINKII**		
246	**HEF zinkii kenneeraa/tetti?** 1=Eeyyee; 2=Lakki-Gara 251 Darbi 3= Ajajuu qofa	|__|
247	**Doozii tokkoon tokkoof 'zinkii ija meeqa? (lakkoofsaan)**	Kinini hanga guyyaati silaa|__|
248	**Guyyaatti 'zinkiin si'a meeqaaf kennamuu qaba?**	Bayina konini al tonnotti fudhatu|__|
249	**Zinkiin guyyaa meeqaaf ajajama?**	Guyyaa |__| fi
250	**HEF akkaataa itti 'zinkii' kennan agarsiiseeraa? (1= Eeyyee; 2= Lakki)**	|__|
251	**HEF warri/kunuunsaan daa'imaa akkaataa 'zinkii'n itti kennamu deebisee akka itti agarsiisu gaafateeraa? (1= Eeyyee; 2= Lakki)**	|__|
252	**HEF warri/kunuunsaan daa'imaa odoo keellaa fayyaatii hin ba'in dura doozii jalqabaa 'Zinkii' daa'imaaf akka kennu gaafateeraa/ofii isaatii kenneeraafii? (1= Eeyyee; 2= Lakki)**	|__|
**VAAYITAAMIN A**		
253	**HEF Vitaami A kenneeraa/tetti?** 1=Eeyyee; 2=Lakki-Gara 259 Darbi 3= Ajajuu qofa	|__|
254	**Vitamin An haala kamiin fudhatamuuf qophaa'e?** (1=50,000 IU kaapsulii; 2=100,000 IU kapsulii; 3=200,000 IU kaapsulii; 8=kan biroo)	|__|
255	**HEF vitamin A kaapsulii meeqa kenne?**	|__| kaapsulii
256	**HEF doozii vitamin A meeqa ajaje/tte?**	|__| doozii /hange
257	**HEF warri/kunuunsaan daa'imaa odoo keellaa fayyaatii hin ba'in dura vitamin A daa'imaaf akka kennu itti himeeraa/ofii isaatii kenneeraafii?** (1=Eeyyee; 2=Lakki)	|__|
258	**HEF vitamin An manatti akka kennamuuf kenneeraa?** (1=Eeyyee; 2=Lakki)	|__|
259	**HEF akkaataa itti 'vitaamin A' kennan agarsiiseeraa? (1= Eeyyee; 2= Lakki)**	|__|

260	**HEF warri/kunuunsaan daa'imaa akkaataa 'vitaamin A' itti kennamu deebisee akka itti agarsiisu gaafateeraa? (1= Eeyyee; 2= Lakki)**	|__|
**PAARASITAAMOOLII**		
261	**HEF paaraasitaamoolii kenneeraa/tetti?**<br>1=Eeyyee; 2=Lakki; 3= Ajajuu qofa	|__|
**AMOOKSAASILIINII**		
262	**HEF amooksaasiliinii kenneeraa?**<br>1=Eeyyee; 2=Lakki-Gara 270tti Darbi 3= Ajajuu qofa	|__|
263	**Amooksaasiliiniin haal kamiin fudhatamuuf qophaa'ee?**<br>**1= liqimsaa; 2= shiroppii; 8= kan biroo**	|__|
264	**Doozii tokkoof Amooksaasiliiniin ija meeqa?**	|__| tablets per dose
265	**Qabiyyeen shirooppii amooksaasiliinii maali/hangami?**<br>**Gaffiin kun kan gafatamuu gaffii 261 =2**	|__|__|__|mg per 5ml
266	**Doozii shirooppiitokkoof amooksaasiliniif ml hangami?**<br>**Gaffiin kun kan gafatamuu gaffii 261 =2**	|__|__| ml per dose
267	**Guyyaatti amooksaasiliiniin si'a meeqaaf kennama?**	|__| times per day
268	**Amooksaasiliiniin guyyaa meeqaaf ajajama?**	For |__| days
269	**HEF akkaataa itti 'amooksaasiliinii' kennan agarsiiseeraa?**<br>**(1= Eeyyee; 2= Lakki)**	|__|
270	**HEF warri/kunuunsaan daa'imaa akkaataa 'amookaasiliinii' itti kennamu deebisee akka itti agarsiisu gaafateeraa?**<br>**(1= Eeyyee; 2= Lakki)**	|__|
271	**HEF warri/kunuunsaan daa'imaa odoo keellaa fayyaatii hin ba'in dura amooksaasiliiniin daa'imaaf akka kennu itti himeeraa/ofii isaatii kenneeraafii?(1= Eeyyee; 2= Lakki)**	|__|
**RUTF**		
272	**HEF RUTF (plaampii natii, BP 100) kenneeraa/kenniteettii?**<br>1=Eeyyee; 2=Lakki-Gara 276tti darbi 3= Ajajuu qofa	|__|
273	**Qabiyyeen RUTF maali dha? (1=plaampii natii; 2= BP 100; 8= kan biroo)**	|__|
274	**Guyyaa tokkoof plaampii natii saakeetii meqa?**	|__| saakeetii guyyaatti
275	**Guyyaa tokkoof BP 100 meeqa?**	|__/ ija guyyaatti
276	**RUTFn guyyaa meeqaaf ajajame?**	Guyyaa |__|
277	**HEF warri/kunuunsaan daa'imaa odoo keellaa fayyaatii hin ba'in dura RUTF daa'imaaf akka kennu gaafateeraa/ofii isaatii kenneeraafii? (1= Eeyyee; 2= Lakki)**	|__|
**YAALII KAN BIROO**		
278	**HEF yaalii kan biro kenneeraa/tetti?**<br>1=Eeyyee; 2=Lakki-Gara 278 darbi 3= Ajajuu qofa-Gara 278 Darbi	|__|
279	**Addeessi**:<br>|____|	
**TALAALLII**		
280	**HEF talaallii kenneeraa/tetti?**<br>1=Eeyyee; 2=Lakki-Gara 280 darbi 3= Ajajuu qofa -Gara 280 darbi	|__|

**281**	**Addeessi**:<br>|____|	
**OL-ERGIINSA**		
**282**	**HEF daa'ima gara dhaabbata fayyaatti ol-ergeeraa/tetti?**<br>(1=Eeyyee; 2=Lakki-Gara 286 darbi)	|__|
**283**	**Warri/kunuunsaan daa'imaa ol-ergiinsa godhame fudhateeraa?**<br>(1=Eeyyee; 2=Lakki)	|__|
**284**	**Sababni ol-ergiinsaa maali ture?**<br>(1=dhukkuba cimaa; 2=qorichi dhibuu; 8=kan biroo (addeessi)	|__|
**285**	**HEF barbaachisummaa ol-erguu ibseeraa/tti?**<br>(1=Eeyyee; 2=Lakki)	|__|
**286**	**HEF yaadannoo ol-ergiinsaa barreesseeraa/tettis?**<br>(1=Eeyyee; 2=Lakki)	|__|
**287**	**Hojjetaan fayyaa geejiba mijeesseeraa/tetti?**<br>(1=Eeyyee; 2=Lakki)	|__|
**GORSA KUNUUNSA MANATTI KENNAMU IRRATTI**		
**288**	**HEF kunuunsa manatti godhamuu qabu irratti gorsa kenneeraa/tetti?**<br>(1=Eeyyee; 2=Lakki)	|__|
**289**	**HEF yoo daa'imni harma hodhuu/dhangala'aa fudhachuu dadhabe, gara dhaabbata fayyaa akka deeman/deebi'anii dhufan gorseeraa/tetti?**<br>(1=Eeyyee; 2=Lakki)	|__|
**290**	**HEF yoo daa'imni dhukkubsate, gara dhaabbata fayyaa akka deeman/deebi'anii dhufan gorseeraa/tetti?** (1=Eeyyee; 2=Lakki)	|__|
**291**	**HEF warri/kunuunsaan daa'imaa dhangala'aa baay'inaan akka kennu gorseeraa/tetti?** (1=Eeyyee; 2=Lakki)	|__|
**292**	**HEF warri/kunuunsaan daa'imaa soruuu akka iitti fufan gorseeraa/tetti?**<br>(1=Eeyyee; 2=Lakki)	|__|
**293**	**HEF warri/kunuunsaan daa'imaa harma hoosisuu/baay'inaa hoosisuu akka iitti fufan gorseeraa/tetti?** (1=Eeyyee; 2=Lakki)	|__|
**294**	**HEF hodoffiif yoom akka deebi'an gorseeraa/tetti?**<br>(1=Eeyyee; 2=Lakki)	|__|
**WANTOOTA HOJIIF BARBAACHISAN/HOJII SALPHISAN**		
**295**	**HEF yeroo daa'ima waliin ture keessatti chaartii (fakkii) ICCMtti fayyadameeraa?** (1=Eeyyee; 2=Lakkki)	|__|
**296**	**HEF yeroo daa'ima waliin ture keessatti galmee ICCMtti fayyadameeraa?**<br>(1=Eeyyee; 2=Lakkki)	|__|

**297**	**Yeroo mariin itti raawwate:**	|__|__|:|__|__|
**298**	**Waliigalaan yeroo daawwiin fudhate herregi**	|__|__| daqiiqaan

DHUMA DAAWWII

Gaaffikkeef deebii si keennuu isaanitiif Warra ykn kunuunsaa daa'imaa galateefachuun gaaffi kamiyyuu yoo qabaatan gaafadhu. Kunuunsaan/warri daa'ima akkaataa itti ORS bulbulamuu, talaalliif yoom deebian, qoricha ajaajame akka itti kennamuu fi yoo daa'imatti dhukkubni itti cime yoom akka deebi'an beekuu isaanii mirkaneeffadhu.
