# Supplementary material for: Quality of clinical assessment and management of sick children by Health Extension Workers in four regions of Ethiopia: A cross-sectional survey
Source: PLoS One. 2020 Sep 25;15(9):e0239361. doi: 10.1371/journal.pone.0239361 (PMC7518593; doi:10.1371/journal.pone.0239361)
Supplement: S1 File — (ZIP) [file pone.0239361.s004.zip › S File/ObservationQuest_Amharic Version.pdf]

የጤና ባለሙያ ዳሰሳ

100	ልዩ የጤና ኤክስቴንሽን ሰራተኛ መ.ቁ (ቀበሌ / ጤና ተቋም / ጤና ኤክስቴንሽን ሰራተኛ ኮድ)	|__|__| / |__|__| / |__|__|
101	ልዩ የህጻን መ.ቁ (ቀበሌ / ጤና ተቋም / ጤና ኤክስቴንሽን ሰራተኛ/ህጻን ኮድ))	|__|__| / |__|__| / |__|__| / |__|__|
102	ቀን	|__||__|/|__||__|/|__||__| ቀን / ወር / ዓ.ም
103	ክልል	|________________________________|
104	ዞን	|________________________________|
105	ወረዳ	|________________________________|
106	ቀበሌ	|________________________________|
107	ክላስተር ኮድ	|__|__|
108	ጂፒ ኤስ ኬክሮስ	|__|__| : |__|__|__|__|__|
109	ጂፒ ኤስ ኬንትሮስ	|__|__| : |__|__|__|__|__|
	ከፍታ	|__|__|__|__|
110	የጤና ድርጅቱ ስም	|________________________________|
111	የጤና ድርጅቱ መለያ ቁጥር /ኮድ	|__|__|
112		
113	የጤና ባለሙያዉ (ጤና ኤክስቴንሽን) መለያ ቁጥር /ኮድ	|__|__|
114	የጤና ባለሙያዉ (ጤና ኤክስቴንሽን) አይነት<br>1=ከ6ኛ ክፍል በታች የትምህርት ደረጃ ያለው ባለሞያ<br>2=ጤና እክስቴነሽን ሰራተኛ	|__|
115	የጤና ባለሙያዉ/ኤክስቴንሽን ሰራተኛ (HW) ጾታ<br>1=ወንድ;<br>2=ሴት	|__|
116_01	የጤና ባለሙያዉ/ኤክስቴንሽን ሰራተኛ መቸ ተወለደ/ v ?	|__||__|/|__||__|/|__||__| ቀን / ወር / ዓ.ም
116_02	የጤና ባለሙያዉ/ኤክስቴንሽን ሰራተኛ የሙያ ªT±?	1= ªT± 1<br>2= ªT± 2<br>3= ªT± 3<br>4= ªT± 4

116_03	**lጤና ኤክስቴንሽን ሙያ Nን ያክል አገለገልሽ/ክ (lለF dlለ የሰራሽውን ¾ምZ)?**	|___||___| *ዐ*L**ት**<br>|___||___| *ወር*
116_04	**lጤና ኤክስቴንሽን ሙያ Nን ያክል አገለገልሽ/ክ (lዝሀ dlለ nu)?**	|___||___| *ዐ*L**ት**<br>|___||___| *ወር*
116_05	**lዝሀ dlለ ነዉ የNrኖርዉ?**	**1 = አዎ 2 = አይደለም**
116_06	**የLኖYያ lr p\saል?**	**1 = አዎ 2 = አል p\ጠኝም**
**የጤና ኤክስቴንሽን ሰራተኛዋ ስልጠና : የአፈጻጸም ግምገማ ሙያዊ ድጋፍ ውይይት ተሳትፎን በተመለከተ**		
**116**	**የተቀናጀ ህ/ሰብ አቀፍ ህጻናት ጤና ህክምና (iCCM) ስልጠና ወስደሃል/ሻል?**<br>**1=አዎ**<br>**2=አልሰለጠንኩም**	|__|
**117**	**የተቀናጀ ህ/ሰብ አቀፍ ህጻናት ጤና ህክምና ስልጠና የወሰደ/ችበት አመት**	|__|__|__|__|
**118**	**ባለፉት 6 ወራት ውስጥ በአፈጻጸም ግምገማ እና ሙያዊ ድጋፍ ውይይት ላይ ተሳትፈሻል ? 1=አዎ**<br>**2=አልተሳተፍኩም**	|__|
**119**	**ባለፉት 6 ወራት ውስጥ የድጋፍና ክትትል ጉብኝት ተደርጎልሻል?**<br>**1=አዎ 2=አልተደረገልኝም**	
**120**	**ለጥያቄ ቁ.120 መልሱ አዎ ከሆነ በቅርቡ ድጋፍ የሰጠው አካል ማ ነው ?**	1=የወረዳ ጤና ጽ/ቤት<br>2=ጤ ና ጣ ቢያ<br>3=መንግስታዊ ያልሆነ ግብረ-ሰናይ ድርጅት<br>4=የወረዳ ጤና ጽ/ቤት እና ጤ ና ጣ ቢያ<br>5=የወረዳ ጤና ጽ/ቤት እና መንግስታዊ ያልሆነ ግብረ-ሰናይ ድርጅት<br>6=ሶስቱ አካላት በአንድነት (የወረዳ ጤና ጽ/ቤት : ጤ ና ጣ ቢያ : መንግስታዊ ያልሆነ ግብረ-ሰናይ ድርጅት)<br>88=ሌላ ግለፅ
**121**	**ለጥያቄ ቁ. 120 መልስዎ አዎ ከሆነ ጉብኝቱ የሚከተሉትን ያጠቃልላል::የሚከተሉትን በሙሉ አንብብ**<br>**ለሁሉም: 1 = አዎ 2 = አይደለም**	
122A	**የሚጠረጠር የሳንባ ምች ምርመራ ወይም ህክምና ላይ የተደረገ ውይይት**	|__|

<table>
<tr><td>122B</td><td>የተቅማጥ በሽታ ምርመራ ወይም ህክምና ላይ የተደረገ ውይይት</td><td>|__|</td></tr>
<tr><td>122C</td><td>የወባ በሽታ ምርመራ ወይም ህክምና ላይ የተደረገ ውይይት</td><td>|__|</td></tr>
<tr><td>122D</td><td>አጭር ግዜ ቆይታ ባለው የምግብ እጥረት በሽታ ምርመራ ወይም ህክምና ላይ የተደረገ ውይይት</td><td>|__|</td></tr>
<tr><td>122E</td><td>የአመዘጋገብ ሁኔታን መመልከት</td><td>|__|</td></tr>
<tr><td>122F</td><td>የህክምና/የጤና አገልግሎት መዝገቦች ባግባቡ/በተሟላ መልክ መመዝገባቸውን ማረጋገጥ</td><td>|__|</td></tr>
<tr><td>122G</td><td>አቅርቦቶችን በተመለከተ ለምሳሌ የስልጠና አጋዥ ማኑዋሎች : የስራ መርጃዎችና የመጠየቅያ ቅፆች መኖራቸውን ማረጋገጥ</td><td>|__|</td></tr>
<tr><td>122H</td><td>የስልጠና አጋዥ ማኑዋሎች : የስራ መርጃዎች:እና የመጠየቅያ ቅፆች አቅርቦት ማድረግ</td><td>|__|</td></tr>
<tr><td>122I</td><td><u>የጤና ኤክስቴንሽን ሰራተኛዋ የህክምና</u> አገልግሎት በምትሰጥበት ግዜ <u>መመልከት</u></td><td>|__|</td></tr>
<tr><td>122J</td><td>የስራ አፈጻጸምን በተመለከተ የጽሁፍ ግብረ-መልስ መስጠት</td><td>|__|</td></tr>
<tr><td>122K</td><td>የተሰጠው ግብረ-መልስ በፅሁፍ ከሆነ : የመጨረሻው ጉብኝት ኮፒ አለ ? (አረጋግጥ)</td><td>|__|</td></tr>
<tr><td>122</td><td>የህጻኑ ስም</td><td>|______________________________|</td></tr>
<tr><td>124</td><td>የህጻኑ መለያ ቁጥር (ኮድ)</td><td>|__|__|</td></tr>
<tr><td>125</td><td>የህጻኑ ጾታ : 1=ወንድ; 2=ሴት</td><td>|__|</td></tr>
<tr><td>126</td><td>የህጻኑ እድሜ ስንት ነው
ከ 1 ዓመት በታች ከሆነ አመት ከ ሚለዉ ቦታ 00 ይጻፉ</td><td>|__|__|አመት
ከ 1 አመት በታች ከሆነ : __|__|ወር</td></tr>
<tr><td>127</td><td>ህጻኑ መቸ ተወለደ?</td><td>|__||__|/|__||__|/|__||__|
ቀን / ወር / ዓ.ም</td></tr>
<tr><td>127_01</td><td>የህጻኑ p ንከባ⁷n ጾታ: 1=ወንድ; 2=ሴት</td><td>|__|</td></tr>
<tr><td>127_02</td><td>ከህጻኑ ¶Y ¦Eው · ን፻}ት?: 1=እናት, 2=አmት, 3=ሌላ (ይጠቀስ)</td><td>|__|</td></tr>
<tr><td>128</td><td>የጤና ኤክስቴንሽንዋ ለgለ መጠይቅ Ùg - ׀¼qለv?</td><td>1=አዎ
2=አልሰጠv ም</td></tr>
<tr><td>129</td><td>የህጻኑ p ንከባ⁷n ለgለ መጠይቅ Ùg - ׀¼qለv/׀ጥቶዋል?</td><td>1=አዎ
2=አልሰጠv ም</td></tr>
<tr><td>130</td><td>የቃለመጠይቅ አቅራቢዉ ስም የመጀመሪያ ፊደላት</td><td>|__|__|</td></tr>
<tr><td>131</td><td>ቀን (ቀን/ወር/አ)ዓ.ም</td><td>|__|__/ |__|__| / |__|__|__|__|</td></tr>
</table>

ሞጁል A: የህጻናት ህክምና ማየት (2 ወር – 5 አመታት)

የህጻኑ ህክምና በትግስት ያለምንም ጣልቃ ገቢነት ይመልከቱ. ያዩትና የሰሙት ይጻፉ.

132 ህክምናውን የተጀመረበት ግዜ ይጻፉ: |__|__|:|__|__|

ዳሰሳ

**ምክንያት**		
	**እናት/ተንከባካቢ ልጁዋን ወደ ጤና ድርጀት ያመጡበት ምንድን ነዉ?** *(መልሱ አዎ ለሚሆኑት ሁሉ 1 ቀጥር ይመዝገብ)*	
133	**የአተነፋፈስ ችግር/ከላይ ከላይ መተንፈስ**	|__|
134	**ሳል**	|__|
135	**የሳንባ ምች**	|__|
136	**ተቅማጥ**	|__|
137	**ትዉከት**	|__|
138	**ትኩሳት**	|__|
139	**ወባ**	|__|
140	**መንዘፍዘፍ/መንቀጥቀጥ**	|__|
141	**የመጠጣት /ጡት የመጥባት ችግር**	|__|
142	**የጆሮ ህመም**	|__|
143	**ሌላ** *(ይጠቀስ)*	|__|
**ክብደት**		
144	**የጤና ኤክስቴንሽን ሰራተኛዋ/ጤና ባለሙያ ወይም ሌላ እስታፍ ዛሬ የህጻኑን ክበደት ለክታ መዝግባለች?** 1=አዎ; 2=የለም	|__|
145	**ተለክቶ ከሆነ ክበደቱ ስንት ነዉ?** (በ ኪሎ ግራም)	|__|__| ኪሎ ግራም
**አደገኛ ምልክቶች**		
146	**የጤና ኤክስቴንሽን ሰራተኛዋ/ጤና ባለሙያዉ ህጻኑ መጠጣት ወይም ጡት መጥባት መቻሉን ወይም አለመቻሉን ጠይቋል?** 1=አዎ; 2=የለም	|__|
146A	**ህጻኑ መጠጣት ወይም ጡት መጥባት አይችልም ?** 1=አዎ; 2=የለም	
147	**የጤና ኤክስቴንሽን ሰራተኛዋ/ጤና ባለሙያዉ ህጻኑ የተመገበውን ሁሉን ነገር ያስታውከዋል ወይ ብሎ ጠይቋል?** 1=አዎ; 2=የለም	|__|
147A	**ህጻኑ የተመገበውን ሁሉን ነገር ያስታውከዋል?**1=አዎ; 2=የለም	|__|
148	**የጤና ኤክስቴንሽን ሰራተኛዋ/የጤና በለሙያዉ ህጻኑ እንደሚያንዘፈዝፈው ጠይቋል?** 1=አዎ; 2=የለም	|__|
148A	**ህጻኑ ያንዘፈዝፈዋል ወይም አሁን እያንዘፈዘፈው ነው ?** 1=አዎ; 2=የለም	|__|
149	**ህጻኑ ሲታይ ንቁ ነዉ? (ይጫወታል ፣ ፈገግ ይላል፣ በደንብ/በሃይል ያለቅሳል)?** 1=አዎ; 2=የለም	|__|
150	**ህጻኑ ሲታይ ንቁ ካለሆነ የጤና ኤክስቴንሽን ሰራተኛዋ/ጤና በለሙያዉ ህጻኑ በጣም አለመዳከሙን/እራሱን አለመሳቱን አረጋግጧል? (ህጻኑ ለ ማንቃት ሞክሯል)** 1=አዎ; 2=የለም	|__|
150A	**ህጻኑ ሲታይ ንቁ ነው/እራሱን አልሳተም ?** 1=አዎ; 2=የለም	|__|
**ሳል / አተነፋፈስ**		

151	**የጤና ኤክስቴንሽን ሰራተኛዋ/ጤና በለሙያዉ ህጻኑ ሳል ወይም የመተንፈስ ችግር እነዳለበት ወይም እነደሌለበት ጠይቋል?** 1=አዎ; 2=የለም	|__|
152	**ህጻኑ ሳል ወይም የመተንፈስ ችግር አለበት?** 1=አዎ; 2=የለም	
**ህጻኑ ሳል ወይም የመተንፈስ ችግር ካለበት ለሚከተሉት ጥያቄዎች (153-157)** 1=አዎ; 2=የለም በማለት መልስ		
153	**የጤና ኤክስቴንሽን ሰራተኛዋ/የጤና ባለሙያዉ ህጻኑ ለምን ያህል ጊዜ እንዳሳለው (የመተንፈስ ችግር እንደገጠመዉ) ጠይቋል ?**	|__|
154	**የጤና ኤክስቴንሽን ሰራተኛዋ/የጤና ባለሙያዉ የህጻኑን አተነፋፈስ ለ 1 ደቂቃ ቆጥራለች/ቆጥሯል?**	|__|
155	**አዎ ከሆነ፣ በ1 ደቂቃ ዉስጥ ምን ያህል ቆጠረች?**	|__|__| የትንፋሽ መጠን በደቂቃ
156	**የጤና ኤክስቴንሽን ሰራተኛዋ/የጤና ባለሙያዉ የደረት ወደ ውስጥ መግባት ስለመኖሩ አይታለች/ቷል?**	|__|
157	**የጤና ኤክስቴንሽን ሰራተኛዋ/የጤና ባለሙያዉ ስትራይደር መኖሩን አይታለች/አዳምጣለች?**	|__|
**ተቅማጥ**		
158	**የጤና ኤክስቴንሽን ሰራተኛዋ/የጤና ባለሙያዉ ህጻኑ ተቅማጥ እንዳለበት ጠይቃለች/ቋል ?** 1=አዎ; 2=የለም	|__|
159	**ህጻኑ ተቅማጥ አለዉ?** 1=አዎ; 2=የለም	|__|
	**ህጻኑ ተቅማጥ ካለዉ *የሚከተሉትን*** (1=አዎ; 2=የለም) በማለት መልስ	
160	**የጤና ኤክስቴንሽን ሰራተኛዋ/የጤና ባለሙያዉ ተቅማጡ ለምን ያህል ጊዜ እንደቆየ ጠይቋል?**	|__|
161	**የጤና ኤክስቴንሽን ሰራተኛዋ/የጤና ባለሙያዉ ተቅማጡ ደም የቀላቀለ እንደነበር ጠይቋል?**	|__|
162	**የጤና ኤክስቴንሽን ሰራተኛዋ/የጤና ባለሙያዉ ሀጻኑ መነጫነጩን ወይም እረፍት ያጣ መሆን አለመሆኑን አለጋግጧል?**	|__|
163	**የጤና ኤክስቴንሽን ሰራተኛዋ/የጤና ባለሙያዉ ለህጻኑ ፈሻሽ ነገር እዲጠጣ ሰጥታለች/ቶታል ?**	|__|
164	**የጤና ኤክስቴንሽን ሰራተኛዋ/የጤና ባለሙያዉ የህፃኑን የሆድ ቆዳ በመቆንጠጥ አይታለች/ቷል?**	|__|
**ትኩሳት**		
165	**የጤና ኤክስቴንሽን ሰራተኛዋ/የጤና ባለሙያዉ ህፃኑ ትኩሳት እንዳለዉ ጠይቃለች/ቋል ወይም በመንካት አረጋግጧለች/ጧል? (ወይም የህጻኑ ሙቀት የተመዘገበ እንደሆነ ተመልከች/ት)** 1=አዎ; 2= የለም	|__|
166	**የጤና ኤክስቴንሽን ሰራተኛዋ/የጤና ባለሙያዉ የህፃኑን የሰዉነት ሙቀነት ለክታለች/ቷል?** 1=አዎ; 2= የለም	|__|
167	**የህፃኑ የሰዉነት ሙቀነት ስንት ሆነ?** (በ ድግሪ ሴንትግሬድ)	|__|__|.|__|ዲግሪ ሴንቲ ግሬድ
168	**ህጻኑ ትኩሳት አለዉ ወይም ባለፉት 48 ሰዓታት ዉስጥ ትኩሳት ነበረዉ?** 1=አዎ; 2= የለዉም	|__|

	**ህጻኑ ትሳት ካለዉ ወይም ከነበረዉ *የሚከተሉትን*** (1=አዎ; 2=የለም) በማለት መልስ	
169	**የጤና ኤክስቴንሽን ሰራተኛዋ/የጤና ባለሙያዉ ህጻኑ ትኩሳቱ ከጀመረዉ ምን ያህል ጊዜ እንደሆነዉ ጠይቃለች/ቋል?**	|__|
170	**የጤና ኤክስቴንሽን ሰራተኛዋ/የጤና ባለሙያዉ ትኩሳቱ በየቀኑ እንደነበር ጠይቃለች/ጠይቋል?**	|__|
171	**የጤና ኤክስቴንሽን ሰራተኛዋ/የጤና ባለሙያዉ ህጻኑ የአንገቱ መገተር መኖር አለመኖሩን አረጋግጣለች/አረጋግጧል?**	|__|
172	**የጤና ኤክስቴንሽን ሰራተኛዋ/የጤና በለሙያዉ የህጻኑ እናቱ ማበጡን እና አለማበጡን አረጋግጣለች/አረጋግጧል?**	|__|
**173**	**የጤና ኤክስቴንሽን ሰራተኛዋ/የጤና በለሙያዉ ህጻኑ በባለፉት 3 ወራት ውስጥ ኩፍኝ እንደነበረው ጠይቃለች/ጠይቋል ወይም የኩፍኝ ምልክቶች መኖራቸዉን እና አለመኖራቸውን አረጋግጣለች/አረጋግጧል?**	**|__|**
174	**የጤና ኤክስቴንሽን ሰራተኛዋ/የጤና በለሙያዉ አጠቃላይ የሰዉነት ሽፍታ ምልክት መኖር አለመኖሩን አረጋግጣለች/አረጋግጧል?**	|__|
175	**ህጻኑ ባለፉት 3 ወራት ጊዜ ውስጥ የኩፍኝ በሽታ ወይም የኩፍኝ ምልክቶች ነበሩት?**	|__|
176	**የጤና ኤክስቴንሽን ሰራተኛዋ/የጤና በለሙያዉ የህጻኑን የአፍ መቁሰል መኖሩን/አለመኖሩን አረጋግጣለች/አረጋግጧል?**	|__|
177	**የጤና ኤክስቴንሽን ሰራተኛዋ/የጤና በለሙያዉ የህጻኑን አይን መግል መሰል ፈሳሽ ወይም ከአይለኑ ብሌን ላይ ነጭ (ደመናማ) ነገር መኖር አለመኖሩን አይታለች/አየቷል?**	|__|
178	**የጤና ኤክስቴንሽን ሰራተኛዋ/የጤና በለሙያዉ ለህጻኑን ለወባ የሚደረግ የፈጣን የደም ምርመራ (አር ዲ ቲ) አድርጋለታለች/አድርጎለታል?**	|__|
179	**የፈጣን የደም ምርመራ/አር ዲ ቲ ምርመራ ከተደረገ፣ ወጤቱ ምን ነበር?** 1=ፖዚቲቭ; 2=ነጋቲቭ; 3=ትክክል ያልሆነ; 9=አላውቅም	|__|
**የጆሮ ህመምን/ችግር በተመለከተ**		
180	**የጤና ኤክስቴንሽን ሰራተኛዋ/የጤና በለሙያዉ ህጻኑን የጆሮ ችግር መኖር አለመኖሩን ጠይቃለች/ጠይቋል?** 1=አዎ 2=የለም	|__|
181	**ህጻኑ/ኗ የጆሮ ችግር አለበት?** 1=አዎ 2=የለም	|__|
	**ህጻኑ የጆሮ ህምም/ችግር ካለበት ...**(1=አዎ; 2=የለም)	
182	**የጤና ኤክስቴንሽን ሰራተኛዋ/የጤና ባለሙያዉ ህጻኑን የጆሮ ህምም/ችግር ከጀመረዉ ስንነት ጊዜዉ እንደሆነው ጠይቃለች/ጠይቋል?**	|__|
183	**የጤና ኤክስቴንሽን ሰራተኛዋ/የጤና ባለሙያዉ ህጻኑ የጆሮ ህመም እንዳለው ጠይቃለች/ጠይቋል?**	|__|
184	**የጤና ኤክስቴንሽን ሰራተኛዋ/የጤና ባለሙያዉ ከህጻኑ ጆሮ ፈሳሽ ነገር ወይም መግል መኖር አለመኖሩን አይታለች/አይቷል?**	|__|
**የተመጣጠነ ምግብ ማነስ**		
185	**የጤና ኤክስቴንሽን ሰራተኛዋ/የጤና ባለሙያዉ የህጻኑን ሁለት እግሮች በመጫን ማበጥ አለማበጣቸዉን አረጋግጣለች/አረጋግጧል?**	|__|

	1=አዎ 2=የለም	
186	**የጤና ኤክስቴንሽን ሰራተኛዋ/የጤና ባለሙያዉ ህጻኑ ግለጽ የሆነ ከፍተኛ የሰውነት መክሳት እንዳለው እና እንደሌለው አይታለች/አይቷል?** 1=አዎ 2=የለም 9=**አይመለከተውም** (NA)	|__|
187	**የጤና ኤክስቴንሽን ሰራተኛዋ/የጤና ባለሙያዉ በላይኛው ክንድ መሀል ላይ በሚለካ የክንድ መጠነ ዙሪያ መለኪያ ለክታለች/ለክቷል?** 1=አዎ 2=የለም 9=**አይመለከተውም** (NA)	|__|
188	**አዎ ካሉ፣ የህጻኑ የላይኛው ክንድ መጠነ ዙሪያ በሴ.ሜ ስንት ነዉ)** 1=(<11); 2=(11.0-11.9); 3=(12.0 **እና ከዚያ በላይ**	|__|
**ደም ማነስ**		
189	**የጤና ኤክስቴንሽን ሰራተኛዋ/የጤና ባለሙያዉ በህጻኑ መዳፍ ላይ ነጭነት ምልክት መኖሩን አይታለች/አይቷል?** 1=አዎ 2=የለም	|__|
**ክትባት**		
190	**የጤና ኤክስቴንሽን ሰራተኛዋ/የጤና ባለሙያዉ የህጻኑ የክትባት ካርድ ለማየት ጠይቃለች/ጠይቋል?** 1=አዎ 2=የለም	|__|
191	**ህጻኑ የክትባት ካርድ አለ?** 1=አዎ 2=የለም	|__|
192	**የጤና ኤክስቴንሽን ሰራተኛዋ/የጤና ባለሙያዉ የህጻኑ የክትባት ሁኔታ/ታሪክ ጠይቃለች/ጠይቋል?** 1=አዎ 2=የለም	|__|
**VITAMIN A**		
193	**የጤና ኤክስቴንሽን ሰራተኛዋ/የጤና ባለሙያዉ ህጻኑ የቫይታሚን ኤ እንክብል ወስዶ ያውቅ እንደሆነ ጠይቃለች/ጠይቋል?** 1=አዎ 2=የለም	

ምደባ

በሽተኞችን ለመመደብ/ለመክፈል የአይ.ሲ.ሲ.ኤም መዝገብ/መጣቀሻ ይጠቀሙ::መደቡ በመመዝገብያ ቅጽ ላይ ካልተመዘገበ **የጤና ኤክስቴንሽን ሰራተኛዋን**/የጤና ባለሞያውን ይጠይቁ::**የጤና ኤክስቴንሽን ሰራተኛዋ/ጤና ባለሙያው** ሁሉንም የበሽተኛ ምደባዎች እስከምትናገር/እስከሚናገር ድረስ "ሌላስ" በማለት ተጨማሪ ምደባ ጠይቅ½ ለእያንዳንዱ ዝርዝር ምደባ አትጠይቅ::

194	**የጤና ኤክስቴንሽን ሰራተኛዋ/ጤና ባለሞያው ለህጻኑ አንድ ወይም ከዚያ በላይ መደብ ሰጥታዋለች/ሰጥቶታል? 1=አዎ; 2=የለም**	|__|
	ከዚህ በታች ባለው ሰንጠረዥ የተሰጡትን ሁሉም ምደባዎች ይመዝገቡ:	
195	**አንድ ወይም ከዚያ በላይ አደገኛ ምልክት (ጡት አለመጥባትም ወይም አለመጠጣት ½ ሁሉም ነገር በተመገበ ቁጥር ማስታወክ ½ መንቀጥቀጥ ½ ሲነካ ብቻ መንቀሳቀስ ወይም ጭራሽ ሲነካም አለመንቀሳቀስ**	|__|
196	**ሀይለኛ የሳንባ ምች/በጣም ሀይለኛ በሽታ**	|__|
197	**የሳንባ ምች**	|__|
198	**የሳንባ ምች የለም**	|__|
199	**ከፍተኛ የሆነ የሰውነት ፈሳሽ ማነስ/የሰውነት መሟሸሽ**	|__|
200	**መካከለኛ የሆነ የሰውነት ፈሳሽ ማነስ/የሰውነት መሟሸሽ**	|__|
201	**የሰውነት ፈሳሽ ማነስ//የሰውነት መሟሸሽ የለም**	|__|
202	**ሀይለኛ የማያቋርጥ ተቅማጥ**	|__|
203	**የማያቋርጥ ተቅማጥ**	|__|
204	**የተቅማጥ በሽታ**	|__|
205	**በጣም ከፍተኛ ትኩሳት ያለው በሽታ**	|__|
206	**ወባ**	|__|
207	**ትኩሳት ½ ወባ መኖሩን የሚያጠራጥር**	|__|
208	**ትኩሳት½ ወባ የለለው**	|__|
209	**የተወሳሰበ ከባድ ኩፍኝ**	|__|
210	**የአይን ወይም የአፍ ኢንፈክሽን ያስከተለ ኩፍኝ**	|__|
211	**ኩፍኝ**	|__|
212	**አዲስ የጆሮ ህመም**	|__|
213	**የቆየ የጆሮ ህመም**	|__|
214	**ከፍተኛ የተመጣጠነ ምግብ እጥረት በሽታ**	|__|
215	**መካከለኛ የተመጣጠነ ምግብ እጥረት በሽታ**	|__|
216	**ከፍተኛ የደም ማነስ**	|__|
217	**የደም ማነስ**	|__|
218	**የክትባት ሁኔታ መረጃው በየጊዜው የተሻሻለ/አብቱዴት አይደለም**	|__|
219a, b	**ሌላ, ግለጽ ....**	|__|
220a, b	**ሌላ, ግለጽ ....**	|__|
221a, b	**ሌላ, ግለጽ ....**	|__|

ህክምና

አስተውል:መረጃ ሰብሳቢው የምክር አገልግሎት በሚሰጥበት ግዜ ስለተደረጉት ምርመራዎችና ስለተሰጡት ህክምናዎች የጤና ኤክስቴንሽን ሰራተኛዋን/ጤና ባለሞያውን መጠየቅ ይችላል።

222	*የጤና ኤክስቴንሽን ሰራተኛዋ*/ጤና ባለሞያው ማንኛውም አይነት ህክምና ወይም ክትባት ሰጥታለች/ሰጥቷዋል? 1=አዎ; 2=የለም	|__|
ኦ.አር.ኤስ		
223	*የጤና ኤክስቴንሽን ሰራተኛዋ*/ጤና ባለሞያው ኦአርኤስ ሰጥታለች/ሰጥታል? 1=አዎ; 2=የለም; 3=እንዲሰጥ አዛለች/አዟል	|__|
224	ስንት የኦአርኤስ እሽግ ተሰጥተዋል? (ቁጥር)	|__|
225	ኦአርኤስ ለመቀበል የምክር አገልግሎት ከተሰጠ በኃላ ህጻኑ በጤና ኬላ እንዲቆይ ተደርጎ ነበር? (1=አዎ; 2=የለም)	|__|
226	*የጤና ኤክስቴንሽን ሰራተኛዋ*/ጤና ባለሞያው ኦአርኤስ እንዴት እንደሚሰጥ በተግባር አሳይታ/ቶ ነበር? (1=አዎ; 2=የለም)	|__|
227	*የጤና ኤክስቴንሽን ሰራተኛዋ*/ጤና ባለሞያው የህጻኑ ተንከባካቢ ኦአርኤስ እንዴት እንደሚሰጡት እንዲያሳዩዋት መልሳ ጠይቃቸው ነበር? (1=አዎ; 2=የለም)	|__|
228	*የጤና ኤክስቴንሽን ሰራተኛዋ*/ጤና ባለሞያው የህጻኑ ተንከባካቢ ከጤና ኬላ ከመሄዱ በፊት የመጀመርያ የኦአርኤስ መጠን እንዲሰጡ ጠይቃለች ወይም ባለሞያው ራሷ ሰጥታለች? (1=አዎ; 2=የለም)	|__|
229	*የጤና ኤክስቴንሽን ሰራተኛዋ*/የጤና ባለሙያው በቤት ውስጥ ስለሚሰጥ ኦ አር ኤስ ፈሳሽ አዛለች/አዝዙዋል? (1. አዎ 2. አላዘዘችም)	|__|
ኮአርተም		
230	የጤና ኤክስቴንሽን ሰራተኛዋ/ጤና ባለሞያው ኮአርተም ትሰጣለች/ይሰጣል? 1=አዎ; 2=የለም; 3=እንዲሰጥ ያዛሉ	
231	በያንዳዱ መጠን/ዶዝ/ ስንት የኮአርተም ክኒን ትሰጣለች/ይሰጣል?	|__| ክኒን በመጠን/በዶዝ
232	ኮአርተም በቀን ስንት ግዜ ይሰጣል?	|__|ግዜ በቀን
233	ኮአርተም ለስንት ቀናት ነው የሚሰጠው/የሚታዘዘው/?	ለ |__| ቀናት
234	ኮአርተም እንደት እንደሚሰጥ የጤና ኤክስቴንሽን ሰራተኛዋ/ጤና ባለሞያው ያሳያሉ? (1=አዎ; 2=የለም)	|__|
235	የጤና ኤክስቴንሽን ሰራተኛዋ/ጤና ባለሞያው የህጻን ተንከባካቢ ኮአርተምን እንዴት ለህጻኑ እንደሚሰጡት እንዲያሳዩአ/ት መልሳ ጠይቃቸዋለች/ጠይቋል? (1=አዎ; 2=የለም)	|__|
236	የጤና ኤክስቴንሽን ሰራተኛዋ/ጤና ባለሞያው የህጻን ተንከባካቢ ከጤና ኬላ ከመሄዱ በፊት የመጀመርያ የኮአርተም መጠን/ዶዝ/ እንዲሰጡ ይጠይቃሉ ወይም ባለሞያው ራሳቸው ይሰጣሉ? (1=አዎ; 2=የለም)	|__|
ኮትሪሞክሳዞል		
237	የጤና ኤክስቴንሽን ሰራተኛዋ/ጤና ባለሞያው ኮትሪሞክሳዞል ሰጣለች/ይሰጣል? 1=አዎ; 2=የለም; 3=እንዲሰጥ ያዛሉ	|__|
238	ያለው የኮትሪሞክሳዞል አዘገጃጀት ምን ይመስላል? 1=የህጻናት ክኒን; 2=የአዋቂዎች ክኒን; 3=ሽሮፕ; 8=ሌላ	|__|
239	ለያንዳዱ መጠን/ዶዝ/ ስንት የኮትሪሞክሳዞል ክኒን ይሰጣሉ? (በቁጥር)	|__|.|__| ክኒን በመጠን/በዶዝ
240	ለያንዳዱ መጠን/ዶዝ/ ስንት የኮትሪሞክሳዞል ሚ.ሊ ሽሮፕ ይሰጣል?	|__| ሚሊ በ ዶዝ

241	ኮትሪሞክሳዞል በቀን ስንት ግዜ ይሰጣል?	|__| ድዜ በቀን
242	ኮትሪሞክሳዞል ለስንት ቀናት ነው የሚሰጠው/የሚታዘዘው/?	ለ |__| ቀናት
243	ኮትሪሞክሳዞል እንዴት እንደሚሰጥ የጤና ኤክስቴንሽን ሰራተኛዋ/የጤና ባለሞያው ያሳያሉ? (1=አዎ; 2=የለም)	|__|
244	የጤና ኤክስቴንሽን ሰራተኛዋ/ጤና ባለሞያው የህጻን ተንከባካቢ ትሪሞክሳዞልን እንዴት እንደሚሰጡት እንዲያሳዩአ/ት መልሳ ትጠይቀዋቸዋል/ይጠይቃቸዋል? (1=አዎ; 2=የለም)	|__|
245	የጤና ኤክስቴንሽን ሰራተኛዋ/ጤና ባለሞያው የህጻን ተንከባካቢ ከጤና ኬላ ከመሄዱ በፊት የመጀመርያ የኮትሪሞክሳዞለ መጠን/ዶዝ/ እንዲሰጡ ይጠየቃሉ ወይም ባለሞያዋ/ው ራሷ/ራሱ ትሰጣለች/ይሰጣል? (1=አዎ; 2=የለም)	|__|
ዚንክ		
246	የጤና ኤክስቴንሽን ሰራተኛዋ/ጤና ባለሞያው ዚንክ ይሰጣሉ? 1=አዎ; 2=የለም; 3=እንዲሰጥ ያዛሉ	|__|
247	ለያንዳዱ መጠን/ዶዝ/ ስንት የዚንክ ክኒን ይሰጣሉ? (በቁጥር)	|__| ክኒን በመጠን/በዶዝ
248	ዚንክ በቀን ስንት ግዜ ይሰጣል?	|__| ግዜ በቀን
249	ዚንክ ለስንት ቀናት ነው የሚሰጠው/የሚታዘዘው/?	ለ |__| ቀናት
250	የጤና ኤክስቴንሽን ሰራተኛዋ/ጤና ባለሞያው ዚንክ እንዴት እንደሚሰጥ ለተንከባካቢው ያሳያሉ? (1=አዎ; 2=የለም)	|__|
251	የጤና ኤክስቴንሽን ሰራተኛዋ/ጤና ባለሞያው የህጻን ተንከባካቢ ዚንክን እንዴት እንደሚሰጡት እንዲያሳዩአ/ት መልሶ ይጠይቀዋቸዋል? (1=አዎ; 2=የለም)	|__|
252	የጤና ኤክስቴንሽን ሰራተኛዋ/ጤና ባለሞያው የህጻው ተንከባካቢ ከጤና ኬላ ከመሄዱ በፊት የመጀመርያ የዚንክ መጠን/ዶዝ/ ባለሞያው ራሳቸው ይሰጣሉ ወይም ተንከባካቢው እንዲሰጡ ይጠይቃሉ? (1=አዎ; 2=የለም)	|__|
ቫይታሚን ኤ		
253	የጤና ኤክስቴንሽን ሰራተኛዋ/ጤና ባለሞያው ቫይታሚን ኤ ይሰጣሉ? 1=አዎ; 2=የለም; 3=እንዲሰጥ ያዛሉ	|__|
254	የቫይታሚን ኤ አዘገጃጀት ምን ይመስላል? (1=50,000 IU ካፕሱል; 2=100,000 IU ካፕሱል; 3=200,000 IU ካፕሱል; 8=ሌላ መጠን)	|__|
255	የጤና ኤክስቴንሽን ሰራተኛዋ/ጤና ባለሞያው ለአንድ ህጻን ስንት የቫይታሚን ኤ ካፕሱል ይሰጣሉ?	|__| ካፕሱልስ
256	የጤና ኤክስቴንሽን ሰራተኛዋ/ጤና ባለሞያው ስንት የቫይታሚን ኤ መጠን/ዶዝ/ ይሰጣሉ/ያዛሉ)	|__| መጠን/ዶዝ/
257	የጤና ኤክስቴንሽን ሰራተኛዋ/ጤና ባለሞያው በጤና ኬላ የቫይታሚን ኤ እንክብል ይሰጣሉ? ወይም የህጻን ተንከባባኪው በጤና ኬላ እንዲሰጥ ይጠየቃሉ? (1=አዎ; 2=የለም)	|__|
258	የጤና ኤክስቴንሽን ሰራተኛዋ/ጤና ባለሞያው በቤት የሚሰጥ ቫይታሚን ኤ ይሰጣሉ?(1=አዎ; 2=የለም)	|__|
259	የጤና ኤክስቴንሽን ሰራተኛዋ/ጤና ባለሞያው ቫይታሚን ኤ እንዴት እንደሚሰጥ ያሳያሉ? (1=አዎ; 2=የለም)	|__|
260	የጤና ኤክስቴንሽን ሰራተኛዋ/ጤና ባለሞያው የህጻን ተንከባካቢ ቫይታሚን ኤን እንዴት ለህጻኑ እንደሚሰጡት እንዲያሳዩአ/ት መልሰው ይጠይቁዋቸዋል? (1=አዎ; 2=የለም)	|__|
ፓራሴታሞል		

261	የጤና ኤክስቴንሽን ሰራተኛዋ/ጤና ባለሞያው ፓራሴታሞል ይሰጣሉ?<br>1=አዎ; 2=የለም; 3=እንዲሰጥ ያዛሉ	|__|
አሞክሳሰሊን		
262	የጤና ኤክስቴንሽን ሰራተኛዋ/ጤና ባለሞያው አሞክሳሰሊን ይሰጣሉ?<br>1=አዎ; 2=የለም; 3=እንዲሰጥ ያዛሉ	|__|
263	የአሞክሳስሊን አዘገጃጀት ምን ይመስላል? 1=ክኒን; 2=ሽሮፕ; 8=ሌላ)	|__|
264	ሌላ ካለ ይጥቀሱ	
265	በያንዳዱ መጠን/ዶዝ/ ስንት የአሞክሳሰሊን ክኒን ይሰጣል?	|__| ክኒን በዶዝ
266	የአሞክሳስሊን ሽሮፕ ጥንካሬ ምን ይመስላል?	|__|__|__|ሚሊ ግራም በ5ሚሊ ሊትር
267	በያንዳዱ መጠን/ዶዝ/ ስንት ሚሊ የአሞክሳስሊን ሽሮፕ ይሰጣል?	|__|__| ሚሊ በዶዝ
268	አሞክሳስሊን በቀን ስንት ግዜ ይሰጣል?	|__| ግዜ በቀን
269	አሞክሳስሊን ለስንት ቀናት ነው የሚሰጠው/የሚታዘዘው/?	ለ |__| ቀናት
270	የጤና ኤክስቴንሽን ሰራተኛዋ/ጤና ባለሞያው አሞክሳሰሊን እንዴት እንደሚሰጥ ለተንከባካቢው ያሳያሉ? (1=አዎ; 2=የለም)	|__|
271	የጤና ኤክስቴንሽን ሰራተኛዋ/ጤና ባለሞያው የህጻን ተንከባካቢ አሞክሳሰሊን እንዴት እንደሚሰጡት እንዲያሳዩአ/ት መልሶ ይጠይቀዋቸዋል? (1=አዎ; 2=የለም)	|__|
272	የጤና ኤክስቴንሽን ሰራተኛዋ/ጤና ባለሞያው የህጻኑ ተንከባካቢ ከጤና ኬላ ከመሄዱ በፊት የመጀመርያ የአሞክሳስሊን መጠን/ዶዝ/ ባለሞያው ራሳቸው ይሰጣሉ ወይም ተንከባካቢው እንዲሰጡ ይጠይቃሉ? (1=አዎ; 2=የለም)	|__|
ፐላምፐይኔት		
273	የጤና ኤክስቴንሽን ሰራተኛዋ/ጤና ባለሞያው ፐላምፐነት (ፐላምፐነት ወይም ቢፒ 1,000) ይሰጣሉ?<br>1=አዎ; 2=የለም; 3==እንዲሰጥ ያዛሉ	|__|
274	የፐላምፐኔት አዘገጃጀት ምን ይመስላል? 1=ፐላምፐኔት; 2=ቢፒ 100; 8=ሌላ	|__|
275	በቀን ስንት ፐላምፐኔት ከረጢት ይሰጣል?	|__| ሳቸት በቀን
276	በቀን ስንት የ ቢፒ 100 ባር ይሰጣል?	|__| ጣሳ በቀን
277	ፐላምፐይኔት/ቢፒ 100 ለስንት ቀን ነው የሚሰጠው?	ለ |__| ቀናት
278	የጤና ኤክስቴንሽን ሰራተኛዋ/ጤና ባለሞያው የህጻኑ ተንከባካቢ ከጤና ኬላ ከመሄዱ በፊት ፐለምፐይነት//ቢፒ 100 ጤና ባለሙያዋ ራሷ ሰጥታቸዋለች ወይም ተንከባካቢው እንዲሰጡ ጠይቆዋል (1=አዎ; 2=የለም)	|__|
ሌሎች ህክምናዎች		
279	የጤና ኤክስቴንሽን ሰራተኛዋ/ጤና ባለሞያው ሌሎች ህክምናዎች ይሰጣሉ?<br>1=አዎ; 2=የለም; 3==እንዲሰጥ ማዘዝ ብቻ	|__|
280	በስም ለይ: |____|	
ክትባቶች		
281	የጤና ኤክስቴንሽን ሰራተኛዋ/ጤና ባለሞያው ክትባቶች ይሰጣሉ?<br>1=አዎ; 2=የለም; 3==እንዲሰጥ ያዛሉ	|__|
282	በስም ለይ: |____|	
ሪፈራል		
283	የጤና ኤክስቴንሽን ሰራተኛዋ/ጤና ባለሞያው ህጻናትን ወደ ጤና ጣቢያ ሪፈር ያደርጋሉ? (1=አዎ; 2=የለም)	|__|

284	**የህጻን ተንከባካቢው የህጻኑን ሪፈራል ይቀበላል? (1=አዎ; 2=የለም)**	|__|
285	**ሪፈር የሚደረግባቸው ምክንያቶች ምንድን ነበር)**<br>**(1=ሀይለኛ ህመም; 2=መድሀኒት ስላለቀ; 8=ሌላ**	|__|
286	**ሌላ ጥቀስ**	
287	**የጤና ኤክስቴንሽን ሰራተኛዋ/ጤና ባለሞያው ሪፈራል ያስፈለገበት ምክንያት አብራርተዋል? (1=አዎ; 2=የለም)**	|__|
288	**የጤና ኤክስቴንሽን ሰራተኛዋ/ጤና ባለሞያው የሪፈራር ወረቀት ጽፈዋል? (1=አዎ; 2=የለም)**	|__|
289	**የጤና ኤክስቴንሽን ሰራተኛዋ/ጤና ባለሞያው የትራንስፖርት አገልግሎት አመቻችተዋል? (1=አዎ; 2=የለም)**	|__|
**በቤት ውስጥ እንክብካቤ ላይ የተሰጠ የምክር አገልግሎት**		
290	**የጤና ኤክስቴንሽን ሰራተኛዋ/ጤና ባለሞያው በቤት ውስጥ ስለሚደረግ እንክብካቤ የምክር አገልግሎት ለተንከባካቢው ይሰጣሉ(1=አዎ; 2=የለም)**	|__|
291	**የጤና ኤክስቴንሽን ሰራተኛዋ/ጤና ባለሞያው ህጻኑ ካልጠጣ ወይም ጡት ካልጠባ ወደ ጤና ተቀም እንዲሄዱ ወይም እንዲመለሱ የምክር አገልግሎት ይሰጣሉ (1=አዎ; 2=የለም)**	|__|
292	**የጤና ኤክስቴንሽን ሰራተኛዋ/ጤና ባለሞያው ህጻኑ የበለጠ ከታመመ ወደ ጤና ተቋም እንዲሄዱ/እንዲመለሱ የምክር አገልግሎት ይሰጣሉ (1=አዎ; 2=የለም)**	|__|
293	**የጤና ኤክስቴንሽን ሰራተኛዋ/ጤና ባለሞያው ተንከባካቢውን ፈሳሽ ነገሮች ጨምረው እንዲሰጡ ይመክራሉ (1=አዎ; 2=የለም)**	|__|
294	**የጤና ኤክስቴንሽን ሰራተኛዋ/ጤና ባለሞያው ተንከባካቢውን ህጻኑን መመገብ እንዲቀጥሉ ይመክራሉ (1=አዎ; 2=የለም)**	|__|
295	**የጤና ኤክስቴንሽን ሰራተኛዋ/ጤና ባለሞያው ተንከባካቢውን ጡት ማጥባት እንዲቀጥሉ ወይም ቶሎ ቶሎ እንዲያጠቡ ይመክራሉ (1=አዎ; 2=የለም)**	|__|
296	**የጤና ኤክስቴንሽን ሰራተኛዋ/ጤና ባለሞያው ለክትትል መቸ እንደሚመለሱ ይመክራሉ (1=አዎ; 2=የለም)**	|__|
**የሥራ መርጃዎች**		
297	**የጤና ኤክስቴንሽን ሰራተኛዋ/ጤና ባለሞያው ከህጻኑ በተገናኘ ቁጥር የ አይሲሲኤም ቻርት መጽሀፍ ትጠቀማለች/ይጠቀማል (1=አዎ; 2=የለም)**	|__|
298	**የጤና ኤክስቴንሽን ሰራተኛዋ/ጤና ባለሞያው ከህጻኑ በተገናኘ ቁጥር የ አይሲሲኤም መመዝገብያ መጽሀፍ ይጠቀማል/ትጠቀማለች (1=አዎ; 2=የለም)**	|__|

299 **የምክክር ግዜ ያለቀበት ሰዓት** |__|__|:|__|__|

300 **ለምልከታ/ትዝብት/ የወሰደውን አጠቃላይ ግዜ አስልተህ አስቀምጥ** |__|__| ደቂቃ

የምልከታ መጨረሻ

ለቃለመጠይቁ አመሰግናለ።
