# Supplementary material for: Quality of clinical assessment and management of sick children by Health Extension Workers in four regions of Ethiopia: A cross-sectional survey
Source: PLoS One. 2020 Sep 25;15(9):e0239361. doi: 10.1371/journal.pone.0239361 (PMC7518593; doi:10.1371/journal.pone.0239361)
Supplement: S1 File — (ZIP) [file pone.0239361.s004.zip › S File/ObservationQuest_English Version.pdf]

Observation Questionnaire: Health Provider Assessment (HPA)

101	**Unique child ID** (kebele / facility / HEW /child code)	|__|__| / |__|__| / |__|__| / |__|__|
102	**Date**	__|__/__|__/|__|__| dd / mm / yy
103	**Region**	|______________________________|
104	**Zone**	|______________________________|
105	**Woreda**	|______________________________|
106	**Kebele**	|______________________________|
107	**Cluster code**	|__|__|
108	**GPS Latitude**	|__|__| |__|__| |__|__| DEG MIN SEC
109	**GPS Longitude**	|__|__| |__|__| |__|__| DEG MIN SEC
	**Elevation**	|______________________________|
110	**Facility name**	
111	**Facility code**	
114	**HEW code**	/__/
115	**HEW type**	1.Under 6 department health worker<br>2.Health extension worker
116	**HEW sex**	1.Male<br>2.Female
116_01	What is the date of birth of HEW?	dd |___|__| mm|___|__| yyyy |___|__|___|__| <br>Ethiopian Calendar
116_02	As an HEW, what is your level?	1 = Level 1<br>2 = Level 2<br>3 = Level 3<br>4 = Level 4
116_03	For how long have you worked as an HEW (including work at other kebeles)? Write number of years.<br>If less than one year, enter number of months only.	|___||___| Years<br>|___||___| Months
116_04	For how long have you worked as an HEW in this Health post? Write number of years and months. If less than one year, enter number of months only	|___||___| Years<br>|___||___| Months
116_05	Do you reside in this kebele?	1 = Yes<br>2 = No
116_06	Was a home provided to you by the kebele?	1 = Yes<br>2 = No
117	**Is the HEW trained in ICCM?**	1=yes;<br>2=no ; If NO skip to 119
118	**Year trained in ICCM**<br>(enter YYYY)	|__|__|__|__|
**119**	**Has the HEW participated on PRCMM during the last 6 months?**	1=Yes<br>2=No
**120**	**Has the HEW received a supportive supervisory visit during the last 6 months?**	1=Yes<br>2=No

**121**	**If yes to 120: Who provided the most recent supervision?**	1=Woreda Health Office<br>2=PHCU/Health Center<br>3=NGO<br>88=Other, Specify
**122**	**If YES to 120**: Did that visit include any of the following? (**Read all the following**<br>**For each 1 = Yes 2 = No**	
**122A**	Discussing diagnosis or treatment of suspected pneumonia	|___|
**122B**	Discussing diagnosis or treatment of diarrhea	|___|
**122C**	Discussing diagnosis or treatment of malaria	|___|
**122D**	Discussing diagnosis or treatment of acute malnutrition	|___|
**122E**	Observing record keeping	|___|
**122F**	Checking the register for consistency and completeness	|___|
**122G**	Checking supplies including training manuals, job aides, request forms	|___|
**122H**	Delivering supplies including training manuals, job aides, request forms	|___|
**122I**	Observing client consultation with HEW	|___|
**122J**	Providing WRTTTEN feedback to you on your work	|___|
**122K**	If WRITTEN feedback: is copy of the last visit available and checked by the interviewer	|___|
123	**Child name**	|______________________________|
124	**Child code**	|__|
125	**Child sex**	1=Male<br>2=Female
126	**How old is [CHILD]?**	124Y.Year____________<br>124M.Month________
127	**Child Birth(dd/mm/yyyy)**	Day____Month______Year________
127_01	**Caregiver's sex**	1=Male<br>2=Female
127_02	**What is the relationship of the caregiver to the child?**	1=Biological mother<br>2=Biological father<br>3=Other, specify
128	***Interviewer*: Has the HEW given consent?**	1=yes<br>2=no. IF NO - END
129	***Interviewer*: Has the caregiver given consent for the consultation to be observed, to complete an exit interview and for the child to be re-examined?**	1=yes<br>2=no. IF NO – END
130	**Interviewer initials**	
131	**Date** (dd/mm/yyyy)	|__|__| / |__|__| / |__|__|__|__|

Module A: Observation Checklist: child (2 months – 5 years)

Observe silently and do not interfere with the consultation. Record what you see and hear.

132 **Time at start of consultation:** |__|__|:|__|__|

Assessment

REASON

	**What reasons does the caregiver give for bringing the child to the health post?** (record 1=yes, 2=no for all that apply)	
133	**Fast/difficult breathing**	1.Yes<br>2.No
134	**Cough**	1.Yes<br>2.No
135	**Pneumonia**	1.Yes<br>2.No
136	**Diarrhea**	1.Yes<br>2.No
137	**Vomiting**	1.Yes<br>2.No
138	**Fever**	1.Yes<br>2.No
139	**Malaria**	1.Yes<br>2.No
140	**Convulsions**	1.Yes<br>2.No
141	**Difficulty drinking or breastfeeding**	1.Yes<br>2.No
142	**Ear problem**	1.Yes<br>2.No
143	**Other (specify)**	|__|
**WEIGHT**		
144	**Does the HEW, or another staff, weigh and record the weight of the child today?**	1=yes<br>2=no ; If NO then skip to 146
145	**What is the child's weight?** (in kg)	|__|__| kg
**DANGER SIGNS**		
146	**Does the HEW ask whether the child is able to drink or breastfeed?**	1=yes<br>2=no
146A	**Is the child unable to drink or breastfeed?**	1=yes; 2=no
147	**Does the HEW ask whether the child vomits everything?**	1=yes<br>2=no; If NO then skip to 148
147A	**Does the child vomit EVERYTHING?**	1=yes; 2=no
148	**Does the HEW ask whether the child has**	1=yes; 2=no

	**convulsions?**	
148A	**Has the child had convulsions or is the child convulsing now?**	1=yes; 2=no
149	**Is the child visibly awake (e.g., playing, smiling, crying with energy)?**	1=yes; 2=no
150	**<u>If child is not visibly awake</u>, does the HEW check for lethargy or unconsciousness (try to wake up the child)?**	1=yes; 2=no
150A	**Is the child unconscious?**	1=yes; 2=no
COUGH / BREATHING		
151	**Does HEW ask for cough or difficult breathing?**	1=yes<br>2=no ; If NO then skip to 154
152	**Does the child have cough or difficult breathing?**	1=yes; 2=no
153	**If child has difficulty breathing: Does the HEW ask how long the child has had cough or difficult breathing?**	1=yes<br>2=no
154	**Does the HEW count breaths in 1 minute?**	1=yes<br>2=no; **If No skip to 156**
155	**If yes, how many breaths does the HEW count in 1 minute?**	|__|__|
156	**Does the HEW look for chest indrawing?**	1=yes<br>2=no
157	**Does the HEW look and listen for stridor?**	1=yes<br>2=no
DIARRHOEA		
158	**Does HEW ask if the child has diarrhea?**	1=yes; 2=no If NO skip to 162
159	**Does the child have diarrhea?**	1=yes<br>2=no
160	**If yes in 159, does the HEW ask how long the child has had diarrhea.**	1=yes<br>2=no
161	**Does the HEW ask if there is blood in the stool?**	1=yes<br>2=no
162	**Does the HEW check if the child is restless or irritable?**	1=yes<br>2=no
163	**Does the HEW offer the child fluid?**	1=yes<br>2=no
164	**Does the HEW pinch the skin of the abdomen?**	1=yes<br>2=no
FEVER		
165	**Does HEW ask/feel for fever (or refer to temperature if taken previously)?**	1=yes<br>2=no
166	**Does the HEW, or another staff, check the temperature of the child?**	1=yes<br>2=no

167	**If checked, what is the child's temperature?** (in degrees)	|__|__|.|__|deg
168	**Does the child have fever or history of fever (last 48 hours)?**	1=yes<br>2=no; If NO skip to 171
169	**Does the HEW ask how long the child has had fever?**	1=yes<br>2=no
170	**Does the HEW ask if the fever has been present every day?**	1=yes<br>2=no
171	**Does the HEW check if the child has a stiff neck?**	1=yes<br>2=no
172	**Does the HEW check if the child has bulged fontanel?**	1=yes<br>2=no
173	**Does the HEW ask if the child has had measles in the last 3 months or check for signs of measles?**	1=yes<br>2=no ; **If NO, skip to 176**
174	**Does the HEW check for generalized rash?**	1=yes<br>2=no
175	**Does the child have history of measles in the last 3 months or signs of measles?**	1.yes<br>2=no
176	**Does the HEW check if the child has mouth ulcers?**	1.yes<br>2=no
177	**Does the HEW look at the child's eyes for pus or clouding of the cornea?**	1.yes<br>2=no
178	**Does the HEW perform an RDT for the child?**	1.yes<br>2=no
179	**If tested with RDT, what is the result?**	1.Postive<br>2.negative<br>3.invalid<br>9.don't know
**EAR PROBLEM**		
180	**Does the HEW ask if the child has an ear problem?**	1=yes<br>2=no; **If NO, skip to 185**
181	**Does the child have an ear problem?**	1=yes<br>2=no
182	**Does the HEW ask how long the child has had an ear problem?**	1=yes<br>2=no
183	**Does the HEW ask if there is ear pain?**	1=yes<br>2=no
184	**Does the HEW check if there is ear discharge/pus draining from the ear?**	1=yes<br>2=no
**MALNUTRITION**		
185	**Does the HEW press on both feet to look for swelling?**	1=yes<br>2=no
186	**Does HEW check for visible severe wasting?**	1=yes

		2=no<br>9 N/A
187	**Does the HEW measure the child's MUAC?**	1=yes<br>2=no<br>9=N/A
188	**If yes, what is the child's MUAC measurement (in cm)?**	1=(<11); 2=(11.0-11.9); 3=(12.0 or above)
**ANAEMIA**		
189	**Does HEW look for palmar pallor?**	1=yes<br>2=no
**VACCINATION**		
190	**Does the HEW ask to see the child's vaccination card?**	1=yes<br>2=no; **If NO, skip to 192**
191	**Is the child's vaccination card available?**	1=yes<br>2=no
192	**Does the HEW ask about the child's vaccination history?**	1=yes<br>2=no
**VITAMIN A**		
193	**Does the HEW ask if the child has ever been given vitamin A capsules?**	1=yes<br>2=no

Classification

Refer to the ICCM register for the patient's classification. If the classification is not recorded in the register ask the HEW what the child's classifications are. Ask "Any other classifications?" until the HEW has stated all classifications. Do not ask for each specific classification.

194	**Does HEW give one or more classifications for the child?**	1=yes<br>2=no; If NO, skip to 222
	Record all classifications given in the table below:	
196	Severe pneumonia/very severe disease	|__|
197	Pneumonia	|__|
198	No pneumonia	|__|
199	Severe dehydration	|__|
200	Some dehydration	|__|
201	No dehydration	|__|
202	Severe persistent diarrhea	|__|
203	Persistent diarrhea	|__|
204	Dysentery	|__|
205	Very severe febrile disease	|__|
206	Malaria	|__|
207	Fever, malaria unlikely	|__|
208	Fever, no malaria	|__|
209	Severe complicated measles	|__|
210	Measles with eye/mouth complications	|__|
211	Measles	|__|
212	Acute ear infection	|__|
213	Chronic ear infection	|__|
214	Severe malnutrition	|__|
215	Moderate malnutrition	|__|
216	Severe anemia	|__|
217	Anemia	|__|
218	Vaccination status not up-to-date	|__|
219	Other, specify ……………………….	|__|
220	Other, specify ……………………….	|__|
221	Other, specify ……………………….	|__|

Treatment

222	**Does HEW administer or prescribe any treatment or vaccine?**	1=yes; 2=no If NO, skip to 280
**ORS**		
223	**Does the HEW give ORS?**	1=yes<br>2=no; **If NO, skip to 229**
224	**How many sachets of ORS are given?** (number)	|__|
225	**Does the HEW recommend that the child stay in the health post after the consultation to receive ORS?**	1=yes<br>2=no
226	**Does the HEW demonstrate how to administer ORS?**	1=yes<br>2=no
227	**Does the HEW ask the caregiver to repeat back how to administer ORS?**	1=yes<br>2=no
228	**Does the HEW give or ask the caregiver to give the first dose of ORS before leaving the health post?**	1=yes<br>2=no
229	**Does the HEW prescribe home-based ORT?**	1=yes<br>2=no
**COARTEM**		
230	**Does the HEW give Coartem?**	1=yes<br>2=no; **If No skip to 237**<br>3.prescribed only
231	**How many Coartem tablets for each dose?**	|__| tablets per dose
232	**How many times should Coartem be given per day?**	|__| times per day
233	**For how many days is Coartem prescribed?**	For |__| days
234	**Does the HEW demonstrate how to administer Coartem?**	1=yes<br>2=no
235	**Does the HEW ask the caregiver to repeat back how to administer Coartem?**	1=yes<br>2=no
236	**Does the HEW give or ask the caregiver to give the first dose of Coartem before leaving the health post?**	1=yes<br>2=no
**COTRIMOXAZOLE**		
237	**Does the HEW give cotrimoxazole?**	1=yes<br>2=no; **If NO, skip to 246**<br>3.prescribed only
238	**What is the formulation of cotrimoxazole?**	1=pediatric tablet; 2=adult tablet; 3=syrup; 8=other
239	**If tablets, how many cotrimoxazole tablets for each dose?** (number)	|__|.|__| tablets per dose
240	**If syrup, how many ml of cotrimoxazole syrup for each dose?**	|__| ml per dose
241	**How many times should cotrimoxazole be given per day?**	|__| times per day
242	**For how many days is cotrimoxazole ~~prescribed~~?**	For |__| days
243	**Does the HEW demonstrate how to administer**	1=yes

	**cotrimoxazole?**	2=no
244	**Does the HEW ask the caregiver to repeat back how to administer cotrimoxazole?**	1=yes<br>2=no
245	**Does the HEW give or ask the caregiver to give the first dose of cotrimoxazole before leaving the health post?**	1=yes<br>2=no
**ZINC**		
246	**Does the HEW give zinc?**	1=yes<br>2=no; **If NO, skip to 253**<br>3.prescirbed only
247	**How many zinc tablets for each dose?**	|__| tablets per dose
248	**How many times should zinc be given per day?**	|__| times per day
249	**For how many days is zinc prescribed?**	For |__| days
250	**Does the HEW demonstrate how to administer zinc?**	1.Yes<br>2.No
251	**Does the HEW ask the caregiver to repeat back how to administer zinc?**	1.Yes<br>2.No
252	**Does the HEW give or ask the caregiver to give the first dose of zinc before leaving the health post?**	1.yes<br>2.No
**VITAMIN A**		
253	**Does the HEW give vitamin A?**	1=yes<br>2=no; **If NO, skip to 261**<br>3=prescribed only
254	**What is the formulation of vitamin A**	1=50,000 IU capsule; 2=100,000 IU capsule; 3=200,000 IU capsule; 8=other)
255	**How many vitamin A capsules does the HEW give?**	|__| capsules
256	**How many doses of vitamin A does the HEW prescribe?**	|__|
257	**Does the HEW give or ask the caregiver to give vitamin A in the health post?**	1=yes<br>2=no
258	**Does the HEW give vitamin A to be given at home?**	1=yes<br>2=no
259	**Does the HEW demonstrate how to administer vitamin A?**	1=yes<br>2=no
260	**Does the HEW ask the caregiver to repeat back how to administer vitamin A?**	1=yes<br>2=no
**PARACETAMOL**		
261	**Does the HEW give paracetamol?**	1=yes;<br>2=no;<br>3=prescribed only
**AMOXICILLIN**		
262	**Does the HEW give amoxicillin?**	1=yes;<br>2=no; **If NO, skip to 273**

		3=prescribed only
263	**What is the formulation of amoxicillin?**	1=tablet;<br>2=syrup<br>8=other
264	**Other, Specify**	
265	**If tablet, how many amoxicillin tablets for each dose?**	|__| tablets per dose
266	**If syrup, what is the strength of the amoxicillin syrup?**	|__|__|__|mg per 5ml
267	**If syrup, how many ml of amoxicillin syrup for each dose?**	|__|__| ml per dose
268	**How many times should amoxicillin be given per day?**	|__| times per day
269	**For how many days is amoxicillin prescribed?**	For |__| days
270	**Does the HEW demonstrate how to administer amoxicillin**	1=yes<br>2=no
271	**Does the HEW ask the caregiver to repeat back how to administer amoxicillin**	1=yes<br>2=no
272	**Does the HEW give or ask the caregiver to give the first dose of amoxicillin before leaving the health post?**	1=yes<br>2=no
**RUTF**		
273	**Does the HEW give RUTF (Plumpy Nut or BP 100)?**	1=yes<br>2=no; **If NO, skip to 279**<br>3=prescribed only
274	**What is the formulation of RUTF?**	1=Plumpy nut; 2=BP100<br>8=other
275	**If Plumpy Nut, how many sachets of Plumpy Nut per day?**	|__| sachets per day
276	**If BP100, how many bars of BP 100 per day?**	|__| bars per day
277	**For how many days is RUTF prescribed?**	For |__| days
278	**Does the HEW give or ask the caregiver to give RUTF before leaving the health post?**	1=yes<br>2=no
**OTHER TREATMENTS**		
279	**Does the HEW give other treatments?**	1=yes;<br>2=no;<br>3=prescribed only
280	**If yes, specify**: |_________________________________|	
**VACCINES**		
281	**Does the HEW give vaccines?**	1=yes;<br>2=no;<br>3=prescribed only
282	**If yes, specify**: |_________________________________|	
**REFERRAL**		
283	**Does the HEW refer the child to a health facility?**	1=yes;

		2=no; **If NO, skip to 290**
284	**Does the caregiver accept referral for the child?**	1=yes<br>2=no
285	**What was the reason for referral?**	1=severe illness<br>2=drug stock out; 8=other (specify)
286	**If other, specify**	
287	**Does the HEW explain the need for referral**	1=yes<br>2=no
288	**Does the HEW write a referral note?**	1=yes<br>2=no
289	**Does the HEW arrange transportation?**	1=yes<br>2=no
**ADVISING ON HOME CARE**		
290	**Does the HEW advise on home care?**	1=yes<br>2=no
291	**Does the HEW advise to go to health facility/return if the child cannot drink or breastfeed?**	1=yes<br>2=no
292	**Does the HEW advise to go to health facility/return if child becomes sicker?**	1=yes<br>2=no
293	**Does the HEW advise caregiver to increase fluids?**	1=yes<br>2=no
294	**Does the HEW advise caregiver to continue feeding?**	1=yes<br>2=no
295	**Does the HEW advise to continue breastfeeding and/or breastfeed more frequently?**	1=yes<br>2=no
296	**Does the HEW advise on when to return for follow-up?**	1=yes 2=no
**JOB AIDS**		
297	**Does the HEW use the iCCM chart booklet at any time during the encounter with the child?**	1=yes<br>2=no
298	**Does the HEW use the iCCM registration book at any time during the encounter with the child?**	1=yes<br>2=no

299	**Time at end of consultation:**	|__|__|:|__|__|
300	**Calculate total time for observation**	|__|__| minutes

"END; THANK THE HEWS AND CARE TAKERS"
