# Supplementary material for: Quality of clinical assessment and management of sick children by Health Extension Workers in four regions of Ethiopia: A cross-sectional survey
Source: PLoS One. 2020 Sep 25;15(9):e0239361. doi: 10.1371/journal.pone.0239361 (PMC7518593; doi:10.1371/journal.pone.0239361)
Supplement: S1 File — (ZIP) [file pone.0239361.s004.zip › S File/ObservationQuest_Tigrigna Version.pdf]

ኣብ ትግበራ ጥሙር ጥዕና ፕሮግራም ዳህሳስ መገልገልቲ ጥዕና

<table>
<tr><td>100</td><td>ብሒቲ መፍለይ ሰራሕተኛ ጥዕና (ጣብያ / ትካል ጥዕና / ኮድ ሰራሕተኛ ጥዕና)</td><td>|__|__| / |__|__| / |__|__|</td></tr>
<tr><td>101</td><td>ብሒቲ መፍለይ ህፃን (ጣብያ / ትካል ጥዕና / ኮድ ህፃን)</td><td>|__|__| / |__|__| / |__|__| / |__|__|</td></tr>
<tr><td>102</td><td>ዕለት</td><td>|__||__|/|__||__|/|__||__|
ዕዕ / ወወ / ዓዓ</td></tr>
<tr><td>103</td><td>ክልል</td><td>|________________________________|</td></tr>
<tr><td>104</td><td>ዞባ</td><td>|________________________________|</td></tr>
<tr><td>105</td><td>ወረዳ</td><td>|________________________________|</td></tr>
<tr><td>106</td><td>ጣብያ</td><td>|________________________________|</td></tr>
<tr><td>107</td><td>ኮድ ተፋሰስ</td><td>|__|__|</td></tr>
<tr><td>108</td><td>ጂፒኤስ ላቲቱድ</td><td>|__|__| : |__|__|__|__|__|</td></tr>
<tr><td>109</td><td>ጂፒኤስ ሎንጊቱድ</td><td>|__|__| : |__|__|__|__|__|</td></tr>
<tr><td>110</td><td>ሽም ጥዕና ትካል ጥዕና</td><td>|________________________________|</td></tr>
<tr><td>111</td><td>ኮድ ትካል ጥዕና</td><td>|__|__|</td></tr>
<tr><td></td><td></td><td></td></tr>
<tr><td>112</td><td>ዓይነት ትካል ጥዕና 1=ማእከል መባእታዊ ክንክን ጥዕና (ማመክጥ) 2=ኬላ ጥዕና</td><td>|__|</td></tr>
<tr><td>113</td><td>ኮድ ሰራሕተኛ ጥሙር ጥዕና</td><td>|__|__|</td></tr>
<tr><td>114</td><td>ዓይነት ሰራሕተኛ ጥዕና 1=ካብ ጥሙር ጥዕና ሰራሕተኛ ወፃኢ ዘሎ ሰራሕተኛ ጥዕና 2=ሰራሕተኛ ጥሙር ጥዕና</td><td>|__|</td></tr>
<tr><td>115</td><td>ፆታ ሰራሕተኛ ጥሙር ጥዕና 1=ተባዕታይ; 2=ኣነስተይቲ</td><td>|__|</td></tr>
<tr><td>116_01</td><td>የጤና ባለሙያዉ/ኤክስቴንሽን ሰራተኛ መቼ ተወለደ/v ?</td><td>|__||__|/|__||__|/|__||__|
ቀን / ወር / ዓ.ም</td></tr>
<tr><td>116_02</td><td>የጤና ባለሙያዉ/ኤክስቴንሽን ሰራተኛ የሙያ ªT±?</td><td>1= ªT± 1
2= ªT± 2
3= ªT± 3
4= ªT± 4</td></tr>
<tr><td>116_03</td><td>Iጤና ኤክስቴንሽን ሙያ Nን ያክል አገለገልሽ/ክ (IለF dIለ የሰራሽውን ¾ምZ)?</td><td>|___||___| 0 L ት
|___||___| ወር</td></tr>
<tr><td>116_04</td><td>Iጤና ኤክስቴንሽን ሙያ Nን ያክል አገለገልሽ/ክ (Iዝህ dIለ n u)?</td><td>|___||___| 0 L ት
|___||___| ወር</td></tr>
<tr><td>116_05</td><td>Iዝህ dIለ ነዉ የN r ኖርዉ?</td><td>1 = አዎ 2 = አይደለም</td></tr>
<tr><td>116_06</td><td>የ L ኖYያ I r p \ s aል?</td><td>1 = አዎ 2 = አል p \ ጠኝም</td></tr>
<tr><td>116</td><td>የጤና ባለሙያዉ/ኤክስቴንሽን ሰራተኛ መቼ ተወለደ/v ?</td><td>|__||__|/|__||__|/|__||__|
ቀን / ወር / ዓ.ም</td></tr>
<tr><td>117</td><td>ጥሙር ጥዕና ሰራሕተኛ ስልጠና ዝተዋደደ ሕብረተሰብ መሰረት ዝገበረ ኣተሓኻኽማ ህፃናትወሲዳ ዶ?
1=እወ; 2=ኣይፋል</td><td>|__|</td></tr>
<tr><td>118</td><td>ስልጠና ዝተወሰድክሉ ዓመት (ኣስፍር ዓዓዓዓ)</td><td>|__|__|__|__|</td></tr>
<tr><td>119</td><td>ባለፉት 6 ወራት ውስጥ የድጋፍና ክትትል ጉብኝት ተደርጎልሻል? 1=አዎ 2=አልተደረገልኝም</td><td></td></tr>
<tr><td>120</td><td>ለጥያቄ ቁ.120 መልሱ አዎ ከሆነ በቅርቡ ድጋፍ የሰጠው አካል ማ ነው ?</td><td>1=የወረዳ ጤና ጽ/ቤት
2=ጤ ና ጣ ቢያ
3=መንግስታዊ ያልሆነ ግብረ-ሰናይ ድርጅት
4=የወረዳ ጤና ጽ/ቤት እና ጤ ና ጣ ቢያ
5=የወረዳ ጤና ጽ/ቤት እና መንግስታዊ ያልሆነ ግብረ-</td></tr>
</table>

<table>
<tr><td></td><td></td><td>ሰናይ ድርጅት
6=ሰስቱ አካላት በአንድነት (የወረዳ ጤና ጽ/ቤት : ጤና ጣ ቢያ : መንግስታዊ ያልሆነ ግብረ-ሰናይ ድርጅት)
88=ሌላ ግለፅ</td></tr>
<tr><td>121</td><td>ለጥያቄ ቁ. 120 መልስዎ አዎ ከሆነ ጉብኝቱ የሚከተሉትን ያጠቃልላል::የሚከተሉትን በሙሉ አንብብ
ለሁሉም: 1 = አዎ 2 = አይደለም</td><td></td></tr>
<tr><td>122A</td><td>የሚጠረጠር የሳንባ ምች ምርመራ ወይም ህክምና ላይ የተደረገ ውይይት</td><td>|__|</td></tr>
<tr><td>122B</td><td>የተቅማጥ በሽታ ምርመራ ወይም ህክምና ላይ የተደረገ ውይይት</td><td>|__|</td></tr>
<tr><td>122C</td><td>የወባ በሽታ ምርመራ ወይም ህክምና ላይ የተደረገ ውይይት</td><td>|__|</td></tr>
<tr><td>122D</td><td>አጭር ግዜ ቆይታ ባለው የምግብ እጥረት በሽታ ምርመራ ወይም ህክምና ላይ የተደረገ ውይይት</td><td>|__|</td></tr>
<tr><td>122E</td><td>የአመዘጋገብ ሁኔታን መመልከት</td><td>|__|</td></tr>
<tr><td>122F</td><td>የህክምና/የጤና አገልግሎት መዝገቦች በግባቡ/በተሟላ መልክ መመዝገባቸውን ማረጋገጥ</td><td>|__|</td></tr>
<tr><td>122G</td><td>አቅርቦቶችን በተመለከተ ለምሳሌ የስልጠና አጋዥ ማኑዋሎች : የስራ መርጃዎችና የመጠየቅያ ቅፆች መኖራቸውን ማረጋገጥ</td><td>|__|</td></tr>
<tr><td>122H</td><td>የስልጠና አጋዥ ማኑዋሎች : የስራ መርጃዎች:እና የመጠየቅያ ቅፆች አቅርቦት ማድረግ</td><td>|__|</td></tr>
<tr><td>122I</td><td><u>የጤና ኤክስቴንሽን ሰራተኛዋ የህክምና </u>አገልግሎት በምትሰጥበት ግዜ <u>መመልከት</u></td><td>|__|</td></tr>
<tr><td>122J</td><td>የስራ አፈጻጸምን በተመለከተ የጽሁፍ ግብረ-መልስ መስጠት</td><td>|__|</td></tr>
<tr><td>122K</td><td>የተሰጠው ግብረ-መልስ በፅሁፍ ከሆነ : የመጨረሻው ጉብኝት ኮፒ አለ ? (አረጋግጥ)</td><td>|__|</td></tr>
<tr><td>122</td><td>የስልጠና አጋዥ ማኑዋሎች : የስራ መርጃዎች:እና የመጠየቅያ ቅፆች አቅርቦት ማድረግ</td><td>|__|</td></tr>
<tr><td>123</td><td>ሽም ህፃን</td><td>|______________________________|</td></tr>
<tr><td>124</td><td>ኮድ ህፃን</td><td>|__|__|</td></tr>
<tr><td>125</td><td>ፆታ ህፃን
1=ተባዕታይ; 2=ኣነስተይቲ</td><td>|__|</td></tr>
<tr><td>126</td><td>ክንደይ ዕድመ ይገብር [ህፃን]?እንድሕር ትሕቲ 1ይ ዓመት ኮይኑ 00 ዓመት ኣዝብል ንኣዋርሕ ብቁፅሪ ፃሓፍ</td><td>|__|__|ዓመት እንድሕር <ዓመትr: |__|__|ኣዋርሕ</td></tr>
<tr><td>127_01</td><td>የህጻኑ p ንከባካn ጾታ: 1=ወንድ; 2=ሴት</td><td>|__|</td></tr>
<tr><td>127_02</td><td>ከህጻኑ ¶Y ¦Eው · ን፻}ት?: 1=እናት, 2=አmት, 3=ሌላ (ይጠቀስ)</td><td>|__|</td></tr>
<tr><td>127</td><td>ዝተወለደሉ ዕለት [ህፃን] መዓዝ እዩ? (ዕለት/ኣዋርሕ/ዓ.ም)</td><td>|__|</td></tr>
<tr><td>128</td><td>ሓታቲ: እቲ ሰራሕተኛ ጥዕና ፍቃድኛ ኮይኑ ዶ?
1=እወ; 2=ኣይፋል. እንድሕር ኣይፋል ኮይኑ – ኣቋርፆ</td><td>|__|</td></tr>
<tr><td>129</td><td>ሓታቲ: ሓብሓቢት ፍቃደኛ ዶ ኮይና ንምዕዛብ፣ ድሕሪ ግልጋሎት ሕቶን ህፃን ዳግም ክምርመርን?
1=እወ; 2=ኣይፋል. እንድሕር ኣይፋል ኮይኑ – ኣቋርፆ</td><td>|__|</td></tr>
<tr><td>130</td><td>መበገሲ ንሓታቲ</td><td>|__|__|</td></tr>
<tr><td></td><td>ዕለት (ዕለት/ወርሒ/ዓመት)</td><td>|__|__| / |__|__| / |__|__|__|__|</td></tr>
</table>

ሞጁል ሀ: መጣለሊ መሕትት ኣብ እዋን ትዕዝቢት: ህፃን (2 ኣዋርሕ – 5 ዓመት ዕድመ)

ትዕዝብትካ ፅሞና ዘለዎን ንዝካየድ ዘሎ ዘተ ዘዕንቅፍ ክኾን የብሉን። እትረአን ትሰምዖን መዝግብ።

131	ዘተ ዝተጀመረሉ ሰዓት:	|__|__|:|__|__|

ዳህሳስ

ምኽንያት		
	ምኽንያት ሓብሓብቲ ንህፃን ናብ ጣብያ ጥዕና/ ኬላ ጥዕና ምምፃኦም? (ይመዝገብ 1=እወ- ንኹሎም ተተግበርቲ)	
132	ቅልጥፍ /መፀገሚ ኣተነፋፍሳ	|__|
133	ታራ ሰዓል/ ጉንፋዕ	|__|
134	ሰንባ ምቺ	|__|
135	ውፅኣት	|__|
136	ተምላስ	|__|
137	ረስኒ	|__|
138	ዓሶ	|__|
139	ምንፍርፋር	|__|
140	ምእባይ ምስታይ ወይ ጡብ ምጥባው	|__|
141	ፀገም እዝኒ	|__|
142	ካልኦት (ይገለፁ)	|__|
ክብደት		
143	ሎማዓንቲ ሰራሕተኛ ጥዕና ወይ ካሊእ ሰራሕተኛ ንህፃን ክብደት ብምምዛን ዝመዝገበ/ት ኣሎ ዶ? 1=እወ; 2=ኣይፋል	|__|
144	ክብደት ህፃን ክንደይ ኮይኑ? (ብኪ/ግራም)	|__|__| ኪ/ግ
ሓደገኛ ምልክታት		
145	ሰራሕተኛ ጥዕና፤ ህፃን ምስታይ ከምዝኽእል ወይ ከምዝጠቡው ሓቲታ ዶ? 1=እወ; 2=ኣይፋል	|__|
146	ሰራሕተኛ ጥዕና፤ ህፃን ኩሉ ብኣፉ ዝወሰዶ ዘምልሶ ንምዃኑ ሓቲታ ዶ? 1=እወ; 2=ኣይፋል	|__|
147	ሰራሕተኛ ጥዕና፤ ህፃን ዘንፈርፍሮ ምህላዉ ሓቲታ ዶ?1=እወ; 2=ኣይፋል	|__|
148	ህፃን ዝረአ ምንቅስቓስ ይገብር ኣሎ ዶ (ኣብነት., ምፅዋት, ሓይሊ ዘለዎ ምስሓቕ, ምብካይ)? 1=እወ; 2=ኣይፋል	|__|
149	እንድሕር እቲ ህፃን ድኹም ምንቅስቓስ ሃልይዎ። ሰራሕተኛ ጥዕና፤ ህፃን ዝልፍልፍ ወይ ምስሓት ውነ ምህላዉ የረጋግፅ? (ህፃን ክንቐሳቐስ ምንቅቓሕ)? 1=እወ; 2=ኣይፋል	|__|
ታራ ሰዓል		
150	ሰራሕተኛ ጥዕና፤ ህፃን ታራ ሰዓል ወይ መፀገሚ ኣተናፋፍሳ ምህላዉ ሓቲታ ዶ? 1=እወ; 2=ኣይፋል	|__|
151	ህፃን ታራ ሰዓል ወይ መፀገሚ ኣተነፋፍሳ ኣለዎ ዶ?1=እወ; 2=ኣይፋል	|__|
	እንድሕር መፀገሚ ኣተነፋፍሳ ሃልይዎ ...	

	(1=እወ; 2=ኣይፋል; ርኡይ ንዝኾነ)	
**152**	ሰራሕተኛ ጥዕና፤ ህፃን ታራ ሳዓል ወይ መፀገሚ ኣተናፋፍሳ ንክንደይ እዋን/ መዓልቲ ክምዝፀንሐ ሓቲታ ዶ?	|__|
**153**	ሰራሕተኛ ጥዕና፤ ህፃን ኣብ ሓደ ደቂቓ በዝሒ ዘተንፎሶ ቆፀራ ዶ?	|__|
**154**	ምላሽ እወ እንተኾይኑ፡ ሰራሕተኛ ጥዕና፤ ህፃን ኣብ ሓደ ደቂቓ በዝሒ ዘተንፎሶ ክንደይ እዩ?	|__|__| በዝሒ ስትንፋስ ኣብ ደቂቓ
**155**	ሰራሕተኛ ጥዕና፤ ህፃን ምስላቅ ትሕቲ ኣፍ ልቢ ኣለዎ ዶ?	|__|
**156**	ሰራሕተኛ ጥዕና፤ ኣብ ህፃን ዝዳመፅ ፀገም ኣተነፋፍሳ/ stridor/ ኣለዎ ዶ?	|__|
ውፅኣት		
**157**	ሰራሕተኛ ጥዕና፤ ህፃን ውፅኣት ምህላዉ ሓቲታ ዶ?<br>1=እወ; 2=ኣይፋል	|__|
**158**	ህፃን ውፅኣት ኣለዎ ዶ?<br>1=እወ; 2=ኣይፋል	|__|
	እንድሕር ውፅኣት ሃልይዎ ... (1=እወ; 2=ኣይፋል)	
**159**	ሰራሕተኛ ጥዕና፤ ህፃን ውፅኣት ንክንደይ እዋን/ መዓልቲ ክምዝፀንሐ ሓቲታ ዶ?	|__|
**160**	ሰራሕተኛ ጥዕና፤ ህፃን ውፅኣት ንምህላዉ እንድሕር ሓቲታ ኣብ ፍታን ደም ኣለዎ ዶ?	|__|
**161**	ሰራሕተኛ ጥዕና፤ ህፃን ውፅኣት ንምህላዉ እንድሕር ሓቲታ ዘይምርግጋእ ወይ ዓወፅዎፅ ኣለዎ ዶ?	|__|
**162**	ሰራሕተኛ ጥዕና ንህፃን ፈሳሲ ህብዎ ዶ?	|__|
**163**	ሰራሕተኛ ጥዕና ንህፃን ምልክት ፃምዕ ሰብነት ንምዕዛብ ቆርበት ከብዱ ቆንጭብዎ ዶ?	|__|
ረስኒ		
**164**	ሰራሕተኛ ጥዕና ህፃን ረስኒ ምህላዉ ሓቲታ/ ወይ ዳህሲሳ ዶ? (ወይ ብመለክዒ ሙቐት ለክዓ እንተፀኒሓ)? 1=እወ; 2=ኣይፋል	|__|
**165**	ሰራሕተኛ ጥዕና ወይ ካሊእ ሰራሕተኛ ሙቐት ህፃን ዝለከዐ/ት ኣሎ ዶ? 1=እወ; 2=ኣይፋል	|__|
**166**	ሙቐት ህፃን ክንደይ እዩ? (ብዲግሪ ሰልሸስ)	|__|__|.|__|
**167**	ህፃን ቅድሚ 48 ሰዓታት ወይ ሐዚ ረስኒ ኣለዎ ዶ? 1=እወ; 2=ኣይፋል	|__|
	እንድሕር ቅድሚ 48 ሰዓታት ወይ ሐዚ ረስኒ ሃልይዎ ...<br>(1=እወ; 2=ኣይፋል; ርኡይ ንዝኾነ)	
**168**	ሰራሕተኛ ጥዕና፤ ህፃን ረስኒ ካብ ዝስሞዖ ክንደይ እዋን/ መዓልቲ ከምዝገበረ ሓቲታ ዶ?	|__|
**169**	ሰራሕተኛ ጥዕና፤ እቲ ረስኒ ኣብ ህፃን ኩሉ መዓልቲ ምንባሩ ሓቲታ ዶ?	|__|
**170**	ሰራሕተኛ ጥዕና፤ ህፃን ክሳድ ምንቃፅ ምህላዉ ሓቲታ ዶ?	|__|
**171**	ሰራሕተኛ ጥዕና፤ ህፃን ምሕባጥ/ ምኹባር መበስበስታ ምህላዉ ሓቲታ ዶ?	|__|
**172**	ሰራሕተኛ ጥዕና፤ ህፃን ኣብ ዝሓለፉ ቅረባ 3ተ ኣዋርሕ ብሕማም ንፍዮ እንተተታሒዙ ወይ ሐዚ ምልክታት ሕማም ንፍዮ ምህላዉ ሓቲታ/ ኣፃርያ ዶ?	|__|
**173**	ሰራሕተኛ ጥዕና፤ ኣብ ህፃን ሓፈሻዊ ሽፍታ ምህላዉ ኣፃርያ ዶ?	|__|
**174**	ህፃን ኣብ ዝሓለፉ ቅረባ 3ተ ኣዋርሕ ታሪክ ሕማም ንፍዮ ወይ ምልክታት ሕማም ንፍዮ ኣለዎ ዶ?	|__|

**175**	ሰራሕተኛ ጥዕና፤ ኣብ ህፃን ኣፍ ቁስለት ምህላዉ ኣፃርያ ዶ?	|__|
**176**	ሰራሕተኛ ጥዕና፤ ኣብ ህፃን ዓይኒ መግሊ ወይ ምሽፋን መርዓት ዓይኒ ምህላዉ ረአ ዶ?	|__|
**177**	ሰራሕተኛ ጥዕና፤ ንህፃን ምርመራ ኣርዲቲ ንሕማም ዓሶ የካይዳ ዶ?	|__|
**178**	እንድሕር ብኣርዲቲ ዝምርምራ ኮይነን ውፅኢት እንታይ ይመስል?<br>1=ኣውንታ; 2=ኣሉታ; 3=ካብ ጥቅሚ ወፃኢ; 9=ኣይፈለጦን	|__|
ፀገም እዝኒ		
**179**	ሰራሕተኛ ጥዕና፤ ህፃን ፀገም እዝኒ ምህላዉ ሓቲታ ዶ? 1=እወ; 2= ኣይፋል	|__|
**180**	ህፃን ፀገም እዝኒ ኣለዎ ዶ? 1=ኣውንታ; 2=ኣሉታ	|__|
	እንድሕር ፀገም እዝኒ ሃልይዎ …<br>(1=እወ; 2=ኣይፋል; ርኡይ ንዝኾነ)	
**181**	ሰራሕተኛ ጥዕና፤ ህፃን ፀገም እዝኒ ንክንደይ እዋን/ መዓልቲ ከምዝገበረ ሓቲታ ዶ?	|__|
**182**	ሰራሕተኛ ጥዕና፤ ህፃን ቃንዛ እዝኒ ከይህልዎ ሓቲታ ዶ?	|__|
**183**	ሰራሕተኛ ጥዕና፤ ህፃን ካብ እዝኒ ዘወፅእ ዝፈስስ ወይ ዝነትዕ መግሊ ምህላዉ ኣፃርያ ዶ?	|__|
ጉድለት ስርዓተ ምግቢ/ሕፅረት ስነመዓዛ		
**184**	ሰራሕተኛ ጥዕና፤ ክልቲኡ እግሪ ህፃን ሕበጥ/ ምሕንባሽ ከይህልዎ ተዓዚባ ዶ?<br>1=እወ; 2=ኣይፋል	|__|
**185**	ሰራሕተኛ ጥዕና፤ ርኡይ ግዱድ ሕብራን ህፃን ምህላዉ ኣፃርያ ዶ? 1=እወ; 2=ኣይፋል	|__|
**186**	ሰራሕተኛ ጥዕና፤ ላዕለዋይ ማእኸላይ ቅልፅም ኢድ ህፃን ለኪዓ ዶ?<br>1=እወ; 2=ኣይፋል	|__|
**187**	እወ እንተኾይኑ, ዓቀን ላዕለዋይ ማእኸላይ ቅልፅም ኢድ ክንደይ እዩ (ብሳ/ሜ)?<br>1=(<11); 2=(11.0-11.9); 3=(12.0 ወይ ልዕሊኡ)	|__|
ዋሕዲ ደም		
**188**	ሰራሕተኛ ጥዕና፤ ከብዲ ኢድ ህፃን ምምንጫሑ ተመለኪቱ ዶ?<br>1=እወ; 2=ኣይፋል	|__|
ክትባት		
**189**	ሰራሕተኛ ጥዕና፤ ክትባት ካርዲ ህፃን ንኽትረአ ሓቲታ ዶ?<br>1=እወ; 2=ኣይፋል	|__|
**190**	ክትባት ካርዲ ህፃን ኣሎ ዶ?<br>1=እወ; 2=ኣይፋል	|__|
**191**	ሰራሕተኛ ጥዕና፤ ኩነታት ታሪክ ክትባት ህፃን ሓቲታ ዶ?<br>1=እወ; 2=ኣይፋል	|__|
ቫይታሚ ኤ		
**192**	ሰራሕተኛ ጥዕና፤ ቫይታሚን ኤ ካፕሱል ህፃን ተዋሂቡዎ ዝፈልጥ ምዃኑ ሓቲታ ዶ?<br>1=እወ; 2=ኣይፋል	|__|

ስያሜ

ንስያሜ ሕሙማት ክሕግዝ መዝገብ ዝተዋደደ ምሕደራ ኣተሓኻኽማ ህፃናት /iCCM/ ተመልከት። እንድሕር ስያሜ ኣብ መዝገብ ዘይሰፈረ ኮይኑ ንሰራሕተኛ ጥዕና ስያሜ ህፃን እንታይ ክኾውን ከምዝኽእል ሕተት።
ሕተት “ካልኦት ስያሜ?” ሰራሕተኛ ጥዕና ኲሎም ስያሜ ክሳብ ዝጥቀሱ ይጥቀሰም። ይኹን እምበር ንሕድ ሕድ ስያሜ ምሕታት ኣየድልን።

**193**	ሰራሕተኛ ጥዕና ንህፃን ክስይም እንተሎ ሓደ ወይ ልዕሊኡ ስያሜ ድዩ ዘቀምጥ?<br>1=እወ; 2=ኣይፋል	|__|
	ኣብ ታሕቲ ዝተዘርዘሩ ኲሎም ስያሜታቶም ኣስፍር:	
**194**	ሓደ ወይ ልዕሊኡ ሓደገኛ ምልክታት (ክሰቲ ዘይምኽኣል ወይ ዘይምጥባው፣ ኲሉ ዝኣተወ ብኣፍ ምምላስ፣ ምንፍርፋር፣ ምንቅስቓስ እንትትንከፍ ወይ እንተተተንከፈ‘ውን ዘይንቀሳቐስ።	|__|
**195**	ከቢድ ሳንባ ምቺ/ ኣዝዩ ከቢድ ሳንባ ምቺ	|__|
**196**	ሳንባ ምቺ	|__|
**197**	ሳንባ ምቺ ዘይኮነ	|__|
**198**	ከቢድ ፃምእ ሰብነት	|__|
**199**	ውስን ፃምእ ሰብነት	|__|
**200**	ፃምእ ሰብነት ዘይኮነ	|__|
**201**	ከቢድ ዝፀንሐ ቅፅኣት	|__|
**202**	ዝፀንሐ ቅፅኣት	|__|
**203**	ደማዊ ውፅኣት	|__|
**204**	ኣዝዩ ከቢድ መራሰንቲ ሕማማት	|__|
**205**	ዓሶ	|__|
**206**	ረስኒ፤ ሕማም ዓሶ ዘይመስል	|__|
**207**	ረስኒ፤ ሕማም ዓሶ ዘይኮነ	|__|
**208**	ከቢድ ዝተሓላለኸ ሕማም ንፍዮ	|__|
**209**	ሕማም ንፍዮ ምስ ሓልክታት ዓይኒ ወይ ኣፍ	|__|
**210**	ሕማም ንፍዮ	|__|
**211**	ቅፅበታዊ ረኽሲ እዝኒ	|__|
**212**	ሕዱር ረኽሲ እዝኒ	|__|
**213**	ከቢድ ሕፅረት ምግቢ	|__|
**214**	ማእኸላይ ሕፅረት ምግቢ	|__|
**215**	ከቢድ ዋሕዲ ደም	|__|
**216**	ዋሕዲ ደም	|__|
**217**	ኲነታት ክታበት ብተኸታታሊ ዘይምውኻታብ	|__|
**218**	ዘድልዩ ክታበት (ይጠቐስ)	|__|
**219**	ቫይታሚ ኤ ብተኸታታሊ ዘይምውሳድ	|__|
**220**	ካልኦት, (ይጠቐስ)	|__|
**221**	ካልኦት, (ይጠቐስ)	|__|
**222**	ካልኦት, (ይጠቐስ)	|__|

ሕክምና

ልብ በል: መፅናዓይ ንሰራሕተኛ ጥዕና ክሕተት ዝደልዮ ምንፃርን ምሕካምን ኣብ እዋን ዘተ ኮይኑ ግና እንድሕር እዞም ክልቲኦም ኣብ ዘተ እዋን ብግልፂ ዘይተቐመጡ እዮም።

**223**	ሰራሕተኛ ጥዕና ክትእዝዞ ዝግባእ ሕክምና ወይ ክታበት ትእዝዝ ዶ?<br>1=እወ; 2=ኣይፋል	|__|
ኦኣርኤስ/ ህይወት መድሕን ንጥረ ነገር		
**224**	ሰራሕተኛ ጥዕና ኦኣርኤስ ትህብ ዶ?1=እወ; 2=ኣይፋል; 3=ምእዛዝ ጥራሕ	|__|
**225**	ክንደይ ዝኣኽሉ ከረጢት ኦኣርኤስ ትህብ? (ብቁፅሪ)	|__|
**226**	ሰራሕተኛ ጥዕና፤ ህፃን ዝተኣዘዘሉ ኦኣርኤስ ክወስድ ኣብ ኬላ ጥዕና ክፀንሕ ትሕብር ዶ? (1=እወ; 2=ኣይፋል)	|__|
**227**	ሰራሕተኛ ጥዕና፤ ኦኣርኤስ ከመይ እሉ ከምዝውሰድ ብተግባር ትሕብር ዶ?<br>(1=እወ; 2=ኣይፋል)	|__|
**228**	ሰራሕተኛ ጥዕና፤ ሓብሓቢት ከመይ ገይራ ኦኣርኤስ ከም ተዳልዎ ዳግም ክተርእያ ትሓታ ዶ? (1=እወ; 2=ኣይፋል)	|__|
**229**	ሰራሕተኛ ጥዕና፤ ሓብሓቢት ቅድሚ ካብ ኬላ ጥዕና ምብጋሳ ፈላማይ ዓቀን ኦኣርኤስ ክትህብ ሓቲታ ዶ? (1=እወ; 2=ኣይፋል)	|__|
**230**	ሰራሕተኛ ጥዕና ገዛ መሰረት ዝገበረ ኣተሓኻኽማ ኦኣርኤስ ትእዝዝ ዶ? (1=እወ; 2=ኣይፋል)	|__|
ኮኣርተም		
**231**	ሰራሕተኛ ጥዕና ኮኣርተም ትህብ ዶ? 1=እወ; 2=ኣይፋል; 3=ምእዛዝ ጥራሕ	
**232**	ሓደ ዓቀን ክንደይ ኮኣርተም ክንን እዩ?	|__| ክንን ሙሉእ ዓቀን
**233**	ኣብ መዓልቲ ክንደይ ግዘ ኮኣርተም ይዋሃብ?	|__| ግዘ ብመዓልቲ
**234**	ኮኣርተም ንክንደይ መዓልቲ ትእዝዝ?	ን |__| መዓልቲታት
**235**	ሰራሕተኛ ጥዕና ኮኣርተም ከመይ ገርካ ከምዝውሰድ ብተግባር ትሕብር ዶ?<br>(1=እወ; 2=ኣይፋል)	|__|
**236**	ሰራሕተኛ ጥዕና፤ ሓብሓቢት ቅድሚ ካብ ኬላ ጥዕና ምብጋሳ ኮኣርተም ከመይ ክዋሃብ ክምዝግቦኦ ክተርእያ ሓቲታ ዶ? (1=እወ; 2=ኣይፋል)	|__|
**237**	ሰራሕተኛ ጥዕና፤ ሓብሓቢት ቅድሚ ካብ ኬላ ጥዕና ምብጋሳ ፈላማይ ዓቀን ኮኣርተም ክትህብ ሓቲታ ዶ? (1=እወ; 2=ኣይፋል)	|__|
ኮትሪሞክሳዞል		
**238**	ሰራሕተኛ ጥዕና ኮትሪሞክሳዞል ትህብ ዶ? 1=እወ; 2=ኣይፋል; 3=ምእዛዝ ጥራሕ	|__|
**239**	ኣደላልዋ ኮትሪሞክሳዞል እንታይ ዓይነት እዩ?<br>1=ህፃናት ክንን ; 2=ዓበይቲ ክንን; 3=ሽሮፕ; 8=ካልኦት	|__|
**240**	ሓደ ዓቀን ክንደይ ኮትሪሞክሳዞል ክንን እዩ? (ብቁፅሪ)	|__|.|__| ክንንያ ብዓቀን
**241**	ሓደ ዓቀን ክንደይ ኮትሪሞክሳዞል ሱሮፕ እዩ?	|__| ኤምኤል ብዓቀን
**242**	ኣብ መዓልቲ ንክንደይ ግዘ ኮትሪሞክሳዞል ይዋሃብ?	|__| ግዘ ብመዓልቲ
**243**	ንክንደይ መዓልቲ ኮትሪሞክሳዞል ይእዘዝ?	ን |__| መዓልቲ
**244**	ሰራሕተኛ ጥዕና ኮትሪሞክሳዞል ከመይ ገርካ ከምዝውሰድ ብተግባር ትሕብር ዶ?<br>(1=እወ; 2=ኣይፋል)	|__|
**245**	ሰራሕተኛ ጥዕና፤ ሓብሓቢት ቅድሚ ካብ ኬላ ጥዕና ምብጋሳ ኮትሪሞክሳዞል	|__|

	ከመይ ክዋሃብ ክምዝግበኦ ክተርእያ ሓቲታ ዶ? (1=እወ; 2=ኣይፋል)	
**246**	ሰራሕተኛ ጥዕና፤ ሓብሓቢት ቅድሚ ካብ ኬላ ጥዕና ምብጋሳ ፈላማይ ዓቀን ኮትሪሞክሳዞል ክትህብ ሓቲታ ዶ? (1=እወ; 2=ኣይፋል)	|__|
ዚንክ		
**247**	ሰራሕተኛ ጥዕና ዚንክ ትህብ ዶ? 1=እወ; 2=ኣይፋል; 3=ምእዛዝ ጥራሕ	|__|
**248**	ሓደ ዓቀን ክንደይ ዚንክ ክንን እዩ? (ብቁፅሪ)	|__| ክንንያ ብዓቀን
**249**	ኣብ መዓልቲ ንክንደይ ግዜ ዚንክ ይዋሃብ?	|__| ግዘ ብመዓልቲ
**250**	ንክንደይ መዓልቲ ዚንክ ይእዘዝ?	ን |__| መዓልቲ
**251**	ሰራሕተኛ ጥዕና ዚንክ ከመይ ገርካ ከምዝውሰድ ብተግባር ትሕብር ዶ? (1=እወ; 2=ኣይፋል)	|__|
**252**	ሰራሕተኛ ጥዕና፤ ንሓብሓቢት ቅድሚ ካብ ኬላ ጥዕና ምብጋሳ ዚንክ ከመይ ክዋሃብ ክምዝግበኦ ክተርእያ ሓቲታ ዶ? (1=እወ; 2=ኣይፋል)	|__|
**253**	ሰራሕተኛ ጥዕና፤ ሓብሓቢት ቅድሚ ካብ ኬላ ጥዕና ምብጋሳ ፈላማይ ዓቀን ዚንክ ክትህብ ሓቲታ ዶ? (1=እወ; 2=ኣይፋል)	|__|
ቫይታሚ ኤ		
**254**	ሰራሕተኛ ጥዕና ቫይታሚ ኤ ትህብ ዶ? 1=እወ; 2=ኣይፋል; 3=ምእዛዝ ጥራሕ	|__|
**255**	ኣደላልዋ ብዓቀን ቫይታሚ ኤ እንታይ እዩ? (1=50,000 IU ካፕሱል; 2=100,000 IU ካፕሱል; 3=200,000 IU ካፕሱል; 8=ካልኦት)	|__|
**256**	ሰራሕተኛ ጥዕና ክንደይ ዝኣክል ቫይታሚ ኤ ካፕሱል ዓቀን ትህብ?	|__| ካፕሱላት
**257**	ሰራሕተኛ ጥዕና ክንደይ ዝኣክል ቫይታሚ ኤ ካፕሱል ዓቀን ትእዝዝ?	|__| ዓቀን
**258**	ሰራሕተኛ ጥዕና፤ ሓብሓቢት ቅድሚ ካብ ኬላ ጥዕና ምብጋሳ ቫይታሚ ኤ ከመይ ክዋሃብ ክምዝግበኦ ክተርእያ ሓቲታ ዶ??(1=እወ; 2=ኣይፋል)	|__|
**259**	ሰራሕተኛ ጥዕና ቫይታሚ ኤ ኣብ ገዛ ክውሰድ ትህብ ዶ?(1=እወ; 2=ኣይፋል)	|__|
**260**	ሰራሕተኛ ጥዕና ቫይታሚ ኤ ከመይ ገርካ ከምዝውሰድ ብተግባር ትሕብር ዶ? (1=እወ; 2=ኣይፋል)	|__|
**261**	ሰራሕተኛ ጥዕና፤ ንሓብሓቢት ቅድሚ ካብ ኬላ ጥዕና ምብጋሳ ቫይታሚ ኤ ከመይ ክዋሃብ ክምዝግበኦ ክተርእያ ሓቲታ ዶ? (1=እወ; 2=ኣይፋል)	|__|
ፓራሲታሞል		
**262**	ሰራሕተኛ ጥዕና ፓራሲታሞል/ፀረ ቃንዛ ትህብ ዶ? 1=እወ; 2=ኣይፋል; 3=ምእዛዝ ጥራሕ	|__|
ኣሞክሳሲሊን		
**263**	ሰራሕተኛ ጥዕና ኣሞክሳሲሊን ትህብ ዶ?<br>1=እወ; 2=ኣይፋል; 3=ምእዛዝ ጥራሕ	|__|
**264**	ኣደላልዋ ኣሞክሳሲሊን እንታይ ዓይነት እዩ? 1=ክንን; 2=ሽሮፕ; 8=other)	|__|
**265**	ሰራሕተኛ ጥዕና ክንደይ ዝኣክል ኣሞክሳሲሊን ዓቀን ትህብ?	|__| ክንንያ ንዓቀን
**266**	ዓቅሚ ኣሞክሳሲሊን ሽሮፕ ከመይ እዩ?	|__|__|__|ሚግ ኣብ 5ኤምኤል
**267**	ሓደ ዓቀን ክንደይ ኣሞክሳሲሊን ኤምኤል ሽሮፕ እዩ?	|__|__| ኤምኤል ብዓቀን
**268**	ሰራሕተኛ ጥዕና ክንደይ ዝኣክል ኣሞክሳሲሊን ዓቀን ኣብ መዓልቲ ትህብ?	|__| ግዘ ኣብ መዓልቲ
**269**	ሰራሕተኛ ጥዕና ንክንደይ መዓልቲ ዝኣክል ኣሞክሳሲሊን ትእዝዝ?	ን |__| መዓልቲ
**270**	ሰራሕተኛ ጥዕና ኣሞክሳሲሊን ከመይ ገርካ ከምዝውሰድ ብተግባር ትሕብር ዶ? (1=እወ; 2=ኣይፋል)	|__|

**271**	ሰራሕተኛ ጥዕና፤ ንሓብሓቢት ኣሞክሳሲሊን ከመይ ክዋሃብ ክምዝግቦእ ክተርእያ ሓቲታ ዶ? (1=እወ; 2=ኣይፋል)	|__|
**272**	ሰራሕተኛ ጥዕና፤ ሓብሓቢት ቅድሚ ካብ ኬላ ጥዕና ምብጋሳ ፈላማይ ዓቀን ኣሞክሳሲሊን ክትህብ ሓቲታ ዶ? (1=እወ; 2=ኣይፋል)	|__|
ኣርዩትኤፍ (ፐላምፐይ ኑት ወይ ቢፒ 100)		
**273**	ሰራሕተኛ ጥዕና ኣርዩትኤፍ (ፐላምፐይ ኑት ወይ ቢፒ 100) ትህብ ዶ?<br>1=እወ; 2=ኣይፋል; 3=ትእዝዝ ጥራሕ	|__|
**274**	ኣደላልዋ ኣርዩትኤፍ እንታይ ዓይነት እዩ? (1=ፐላምፐይ ኑትt; 2=ቢፒ100; 8=ካልኦት)	|__|
**275**	ኣብ መዓልቲ ክንደይ ዝኣክል ሳኬት ፐላምፐይ ኑት ትህብ?	|__| ሳኬት ኣብ መዓልቲ
**276**	ኣብ መዓልቲ ክንደይ ዝኣክል ሳኬት ባርስ ቢፒ 100 ትህብ?	|__| ባርስ ኣብ መዓልቲ
**277**	ሰራሕተኛ ጥዕና ንክንደይ መዓልቲ ዝኣክል ኣርዩቲኤፍ ትእዝዝ?	ን |__| መዓልቲ
**278**	ሰራሕተኛ ጥዕና፤ ንሓብሓቢት ቅድሚ ካብ ኬላ ጥዕና ምብጋሳ ኣርዩትኤፍ ከመይ ክዋሃብ ክምዝግቦእ ክተርእያ ሓቲታ ዶ? (1=እወ; 2=ኣይፋል)	|__|
ካልኦት ሕክምና		
**279**	ሰራሕተኛ ጥዕና ካልኦት ሕክምና ትህብ ዶ?<br>1=እወ; 2=ኣይፋል; 3=ትእዝዝ ጥራሕ	|__|
**280**	ይጠቀሱ:<br>|____|	
ክታበት		
**281**	ሰራሕተኛ ጥዕና ክታበት ትህብ ዶ?<br>1=እወ; 2=ኣይፋል; 3=ትእዝዝ ዶ	|__|
**282**	ይጠቀሱ:<br>|____|	
ቅብብል ሕሙማት		
**283**	ሰራሕተኛ ጥዕና ንህፃን ናብ ብልዕሊኣ ትካል ጥዕና ትሰድድ ዶ? (1=እወ; 2=ኣይፋል)	|__|
**284**	ሓብሓቢት ቅብብል ሕሙም ንህፃን ቅቡል ትገብሮ ዶ? (1=እወ; 2=ኣይፋል)	|__|
**285**	ምኽንያት ቅብብል ሕሙም ህፃን እንታይ እዩ?<br>(1=ከቢድ ሕማም; 2=ፈውሲ ብምውዳኡ; 8=ካልኦት (ይጠቀስ)	|__|
**286**	ሰራሕተኛ ጥዕና ኣዳላይነት ቅብብል ሕሙም ገለፃ ትገብር ዶ? (1=እወ; 2=ኣይፋል)	|__|
**287**	ሰራሕተኛ ጥዕና ስሩዕ ቅብብል ሕሙማት ይፅሕፍ ዶ? (1=እወ; 2=ኣይፋል)	|__|
**288**	ሰራሕተኛ ጥዕና መጓዓዓዚ የመዓራሪ ዶ? (1=እወ; 2=ኣይፋል)	|__|
ምምክር ኣብ ገዛ ዝዋሃቡ ክንክን		
**289**	ሰራሕተኛ ጥዕና ኣብ ገዛ ዝግበሩ ክንክን ምክሪ ይህብ ዶ? (1=እወ; 2=ኣይፋል)	|__|
**290**	ሰራሕተኛ ጥዕና ህፃን ናብ ትካል ጥዕና ክኸድ/ ህፃን ምስታይ ወይ ምጥባው ተዘይክእሉ ተመሊሱ ክመፅእ ምኽሪ ሂባ ዶ? (1=እወ; 2=ኣይፋል)	|__|
**291**	ሰራሕተኛ ጥዕና ህፃን ናብ ትካል ጥዕና ክኸድ/ ህፃን እንተተፀጊዑዎ ተመሊሱ ክመፅእ ምኽሪ ሂባ ዶ? (1=እወ; 2=ኣይፋል)	|__|
**292**	ሰራሕተኛ ጥዕና ንሓብሓቢት ተወሳኺ ፈሳሲ ክትህብ ምክሪ ሂባ ዶ? (1=እወ; 2=ኣይፋል)	|__|
**293**	ሰራሕተኛ ጥዕና ንሓብሓቢት ኣመጋግባ ክቅፅል ምክሪ ሂባ ዶ? (1=እወ; 2=ኣይፋል)	|__|
**294**	ሰራሕተኛ ጥዕና ብቐፃሊ ፀባ ጡብ ኣዶ ብተደጋጋሚ ክጠቡው ምክሪ ሂባ ዶ? (1=እወ; 2=ኣይፋል)	|__|

**295**	ሰራሕተኛ ጥዕና፤ ሓብሓቢት ንክትትል መዓዝ ክትምለስ ከምዘለዋ ምክሪ ሂባ ዶ? (1=እወ; 2=ኣይፋል)	|__|
መምሀሪ ሓገዝ		
**296**	ሰራሕተኛ ጥዕና ኣብ እዋን ምርመራ ህፃን ዝተዋደደ ሕብረተሰብ ምሕደራ ኣተሓኻኽማ ህፃናት/ iCCM/ ቻርት ቡክ ሊት ትጥቀም ዶ? (1=እወ; 2=ኣይፋል)	|__|
**297**	ሰራሕተኛ ጥዕና ኣብ እዋን ምርመራ ህፃን ዝተዋደደ ሕብረተሰብ ምሕደራ ኣተሓኻኽማ ህፃናት መዝገብ ትጥቀም ዶ? (1=እወ; 2=ኣይፋል)	|__|
**298**	መሕትት ዝተወደአሉ ግዘ:	|__|__|:|__|__|
**299**	ምግባጥ ንትዕዝቢት ጠቅላላ ዝወዳ ግዘ	|__|__| ብደቂቓ

ፍፃመ ትዕዝቢት

ሓብሓብቲ ንዝሓተትኩኹም ዝሃብኩምኒ ምላሽ የመስግን። ዝኾነ ሕቶ እንተሃልይኩን ወይ እንተሃልዩኩም ሕተታኒ/ ሕተቱኒ። ኣረጋጊፂ ሓብሓብቲ ውፅኣት ንዘለዎ ህፃን ኣደላልዋ ሂወት መድሕን ንጥረ ነገር፣ ንክትባት መዓዝ ከምትምለስ፣ እቲ ዝተኣዘዘ ፈውሲ ከመይ ከምዝውሰድን ህፃን ኣብ ገዛ እንተተፀኒዕዎ መዓዝ ከምትምለስ
